# H_2_O_2_ Production via Artificial Photosynthesis Over Defective Graphitic Carbon Nitride

**DOI:** 10.1002/advs.202507913

**Published:** 2025-08-12

**Authors:** Yiwen Zhang, Dingle Wu, Xiaofei Zeng, Bocheng Qiu, Qiaohong Zhu, Jinlong Zhang

**Affiliations:** ^1^ Key Laboratory of Organosilicon Chemistry and Material Technology Ministry of Education Zhejiang Key Laboratory of Organosilicon Material Technology College of Material Chemistry and Chemical Engineering Hangzhou Normal University Hangzhou Zhejiang 311121 China; ^2^ Jiangsu Key Laboratory of Pesticide Sciences Department of Chemistry College of Sciences Nanjing Agricultural University Nanjing 210095 China; ^3^ State Key Laboratory of Green Chemical Engineering and Industrial Catalysis School of Chemistry and Molecular Engineering East China University of Science & Technology Shanghai 200237 China

**Keywords:** defective carbon nitride, H_2_O_2_ photosynthesis, in situ characterization, in‐deep mechanism investigation, structure‐activity relationship

## Abstract

Metal‐free graphitic carbon nitride has been considered as a promising candidate for hydrogen peroxide (H_2_O_2_) photosynthesis, with the advantage of low‐cost, high stability, and environmentally friendly capacity. However, such a solar‐to‐chemical conversion still suffers from limited light utilization, confined surface adsorption, and restricted reduction/oxidation reaction pathway. At this juncture, this bottleneck has been overcome through diverse modifications over carbon nitride, such as defect engineering graphitic carbon nitride with modulated physical and chemical properties, which performs satisfactory visible‐light absorption, sufficient active sites, and promotes charge transfer kinetics for artificial solar‐to‐chemical conversion. In this review, the recent advances for H_2_O_2_ photosynthesis over defective graphitic carbon nitride are described, including the existing principles for H_2_O_2_ formation, the factors affecting photosynthesis, the fabrication strategies toward novel materials, and the detailed pathways for H_2_O_2_ formation. The functions of defects, the properties of materials, and the reaction mechanisms, and the selectivity, as well as in situ characterizations for catalyst synthesis and pathway exploration, have been summarized clearly. Finally, the advantages and shortcomings, together with the challenges and prospects, are highlighted for the development of defect engineering over carbon nitride for H_2_O_2_ photosynthesis.

## Graphical abstract

The emerging unique properties of defective graphitic carbon nitride endow it as a promising candidate for H_2_O_2_ photosynthesis. This review aims to offer a full‐scale lens to discuss the recent advances of defective carbon nitride, covering band structure engineering, electronic structure analysis, pathway regulation, and in situ technologies, and envision the prospects of development in the future.

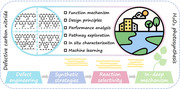

## 1 Introduction

Photocatalytic H_2_O_2_ generation has emerged as a robust tool to deal with the incessant environmental issues, necessitating the discovery of preeminent photocatalysts.^[^ ^1^, ^2^, ^3^, ^4^ ^]^ Metal‐free graphitic carbon nitride (g‐C_3_N_4_), as a compelling star with a unique structure, offers a desirable pathway for solar‐to‐chemical conversion, owing to its properties with an easy‐fabrication process and intriguing light utilization.^[^ ^5^, ^6^, ^7^ ^]^ Typically, it has been investigated that the minimum conduction band (CB) and maximum valance band (VB) of carbon nitride might be −1.33 and 1.44 V, with an ideal bandgap of 2.77 eV,^[^ ^8^ ^]^ exhibiting opportune visible light absorption, which makes it propitious for general photocatalytic reaction, including water splitting,^[^ ^9^, ^10^, ^11^, ^12^ ^]^ carbon dioxide (CO_2_) reduction,^[^ ^13^, ^14^, ^15^ ^]^ H_2_O_2_ formation,^[^ ^16^, ^17^ ^]^ nitrogen (N_2_) fixation,^[^ ^18^, ^19^ ^]^ wastewater treatment,^[^ ^20^, ^21^ ^]^ plastic disposal,^[^ ^22^, ^23^, ^24^, ^25^ ^]^ chemical organic synthesis,^[^ ^26^ ^]^ *etc*. Thereinto, recent advances of carbon nitride for H_2_O_2_ photosynthesis have riveted the attention of researchers, by virtue of the noteworthy properties of carbon nitride for oxygen reduction and water oxidation.^[^ ^4^, ^27^ ^]^ Nevertheless, some imperative issues and limitations have emerged and impeded the development of g‐C_3_N_4_, like limited solar energy utilization, inadequate surface area and active sites, suppressed molecules adsorption and activation, and unsatisfied charge separation efficiency.

Concerning these issues, diverse strategies have been investigated for the modification of carbon nitride, that is, defect engineering,^[^ ^28^, ^29^, ^30^ ^]^ morphology design,^[^ ^31^, ^32^, ^33^ ^]^ heterojunction formation,^[^ ^34^, ^35^ ^]^ co‐catalysts introduction,^[^ ^36^, ^37^ ^]^ *etc*., in which defect engineering is deemed as a salient means for developing desirable carbon nitride for H_2_O_2_ photosynthesis. Defect engineering strategy, including element doping, vacancies addition, and functional formation, *etc*., have been explored over g‐C_3_N_4_ for photocatalytic reactions, with modulated electronic structures, adjustable band structures, extended light utilization, and accelerated charge transfer.^[^ ^38^, ^39^, ^40^, ^41^ ^]^ Concomitantly, the increased surface area and active sites lay the foundation for a strengthened adsorption capacity of the catalyst, which is vital for solar‐to‐chemical photosynthesis. For instance, a holey defective g‐C_3_N_4_ photocatalyst was developed by Ye’ group^[^ ^42^ ^]^ for visible‐light‐driven H_2_O_2_ production, with the narrowed bandgap for extended light absorption and the formation of defect states within the bandgap for the suppression of charge recombination. Taking another example, Wang's group^[^ ^43^ ^]^ synthesized a homo‐junction with multiple order–disorder interfaces for H_2_O_2_ photosynthesis with a superior two‐step single‐electron oxygen reduction reaction process, attributed to the interfacial engineering with more active sites, the strengthened light absorption, as well as the established internal electric field (IEF) for electron migration. Nevertheless, the existence of defects might also perform as the recombination centers of charge carriers, which hamper photo‐generated electrons and holes transfer and separation.^[^ ^44^ ^]^ Thereafter, understanding the formation methods and the function of defects over g‐C_3_N_4_ for H_2_O_2_ photosynthesis is indispensable. Notably, the introduction of defects into the structure of g‐C_3_N_4_ might enhance photocatalytic performance and reaction selectivity through optimizing band structures, modulating electronic structures, accelerating charge transfer, and strengthening surface adsorption capacity.^[^ ^45^, ^46^ ^]^ The formation of defects in g‐C_3_N_4_ leads to the appearance of thermodynamically unstable coordination‐unsaturated atoms, which are favorable for the adsorption and activation of reactant molecules.^[^ ^47^ ^]^ However, the presence of defects might also lead to some undesired effects, which need to be taken into consideration in practical applications. For instance, to precisely control the type, concentration, and distribution of defects, complex preparation processes and accurate control conditions are required.^[^ ^48^ ^]^ Although defects can suppress the recombination of photogenerated charge carriers to some extent, an excessive number of defects or improper types of defects might lead to the formation of undesired recombination sites for photogenerated electrons and holes, thereby reducing photocatalytic efficiency.^[^ ^49^ ^]^ Thereinto, the advantages and limitations of defective carbon nitride are also summarized and discussed thoroughly in this review.

In parallel to consistent investigations in the realm of defect‐engineering carbon nitride, several reviews have been reported concerning the modification of g‐C_3_N_4_ or its efficiency for photocatalytic H_2_O_2_ production in recent years. For example, Zhang's group^[^ ^45^ ^]^ displayed recent progress concerning engineering defects in graphitic carbon nitride photocatalysts, and summarized the performance and mechanism for varying applications, including photocatalytic H_2_ evolution, CO_2_ reduction, N_2_ fixation, and organic transformations, providing a deep understanding of defects in various applications. In another case, Lei and his co‐workers^[^ ^8^ ^]^ concluded the energy band engineering of graphitic carbon nitride for photocatalytic hydrogen peroxide production and discussed recent advances of carbon nitride for H_2_O_2_ photosynthesis, thus providing a guidance and conclusion referring the inherent relationship between the control strategies and their energy band structure. Despite such impressive achievements, a comprehensive review covering not only the construction of defects but also its functions and internal mechanisms for H_2_O_2_ synthesis is missing, however, of huge necessity.

**Figure 1:**
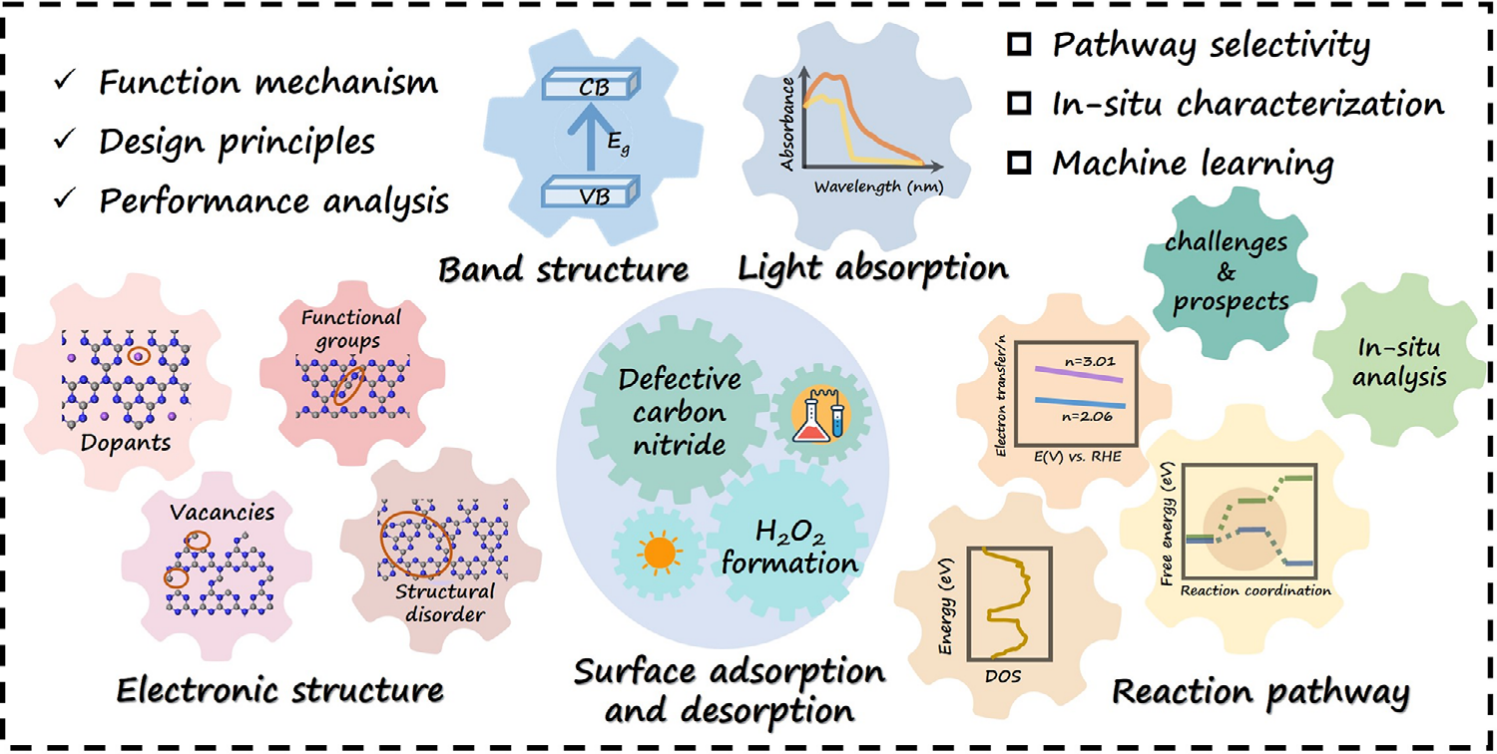
A summative illustration of overall contents concerning defective graphitic carbon nitride for H_2_O_2_ photosynthesis.

In this review, the recent process of defect‐engineering carbon nitride for H_2_O_2_ photosynthesis are lay out on five parts (**Figure** **1**): i) the synthetic strategies for diverse defect‐engineering g‐C_3_N_4_, the function mechanism and design principles of defects; ii) band structures engineering and its influences for light absorption; iii) electronic structure modulation with strengthened surface adsorption capacity for redox reactions; iv) structure‐activity interactions and DFT calculations over the pathways for H_2_O_2_ photosynthesis; v) in situ characterizations in recent researches with in‐deep exploration. Finally, the challenges and prospects about the development of defect‐engineering carbon nitride for solar‐to‐chemical conversion over H_2_O_2_ are pointed out.

## 2 Design Principles of Defective g‐C3N4 for H_2_O_2_ Photosynthesis

It is well acknowledged that photocatalytic H_2_O_2_ formation can be divided into three steps: light utilization for electrons‐holes pairs generation, surface adsorption and photo‐generated charge carriers migration, and charge‐carrier‐induced surface redox reactions.^[^ ^50^, ^51^ ^]^ Within this scenario, light absorption, surface adsorption, and charge separation are vital for the photocatalytic system, which are essential prerequisites for realizing satisfactory performance. In light of this, defect‐modified g‐C_3_N_4_ with modulated electronic and band structures is usually considered as a near‐ideal candidate for solar‐to‐chemical conversion. Defect engineering, including vacancy formation, atomic dopants, functional group incorporation, and structural disorder, has been extensively investigated and is laid out here (**Table** **1**). Essentially, the synthetic process for diverse defects and the intrinsic reaction pathway are of vital importance, but different when defects vary. Herein, some general synthetic strategies for defect introduction are concluded, the basic functions and the design principles are listed.

**Table 1:** Synthetic strategies for various types of defects.

Photocatalysts	Synthetic method	type of defect	Refs.
Cv‐g‐C_3_N_4_	calcinations with Ar	C defects	[52]
CNH^a)^	water‐activated method	C defects	[53]
Cv‐TCN^b)^	thermal treatment	C defects	[54]
PCNC^c)^	self‐template	C defects and O doping	[55]
g‐C_3_N_4_‐N_3C_	In situ‐copyrolysis	N defects	[56]
CKCN‐0.03^d)^	KOH‐assisted calcination treatment	N defects	[57]
g‐C_3_N_4_‐ND4‐OM3^e)^	calcination and acid‐etching	N defects and O dopants	[58]
CNP_0.6_ ^f)^	hydrothermal strategy	N defects and O‐containing defects	[59]
KCN_x_	one‐pot synthesis approach	N defect and K doped	[60]
D‐TCN_450_ ^g)^	Self‐supramolecular reaction	N defects and boron dopants	[61]
SD‐CN^h)^	fusion polymerization	N defects and Na doping	[62]
g‐C_3_N_4_‐U^i)^	thermal polymerization	N defects and O dopants	[63]
bmw‐DCN‐x^j)^	template	N defects and Na doping	[64]
U/AC_0.5_ ^k)^	thermal polymerization	N defects and O doping	[65]
V_N_‐CNOH‐45	self‐assemble	N defects and hydroxyl groups	[54]
A‐V‐g‐C_3_N_4_ ^l)^	thermal treatment	N defects and ─C ≡ N	[66]
CNQ680^m)^	thermal treatment	N defects and ─C ≡ N	[67]
D‐CNNS	solid‐state copolymerization	bridging‐nitrogen defects	[68]
DCN‐m^n)^	molten salt assisted post calcining method	─C ≡ N defects	[69]
TCNS^o)^	thermal condensation	introduced ─C ≡ N groups	[70]
SDCN	thermal polymerization	S doping and ─C ≡ N	[71]
MTCN^p)^	thermal polymerization	S doping and ─C ≡ N	[20]
SCN‐x^q)^	unstable organic frameworks	O doping	[72]
CN‐NH_4_‐NaK	soft template strategy	order–disorder interfaces	[43]
CACN^r)^	two‐step adjacent calcination strategy	order–disorder interfaces	[16]

^a)^ CNH: porous g‐C_3_N_4_ with carbon vacancies by co‐pyrolysis of water and melamine;

^b)^ Cv‐TCN: porous carbon nitride nanotube (TCN) photocatalysts with abundant carbon vacancies (Cv);

^c)^ PCNC: oxygen‐doped carbon nitride tubes with a pipe‐in‐pipe double‐layer (PCN) and carbon defects;

^d)^ CKCN‐0.03: carbon nitride by calcining with 0.03 g KOH;

^e)^ g‐C_3_N_4_‐ND4‐OM3: oxygen‐modified graphite carbon nitride with nitrogen‐defect;

^f)^ CNP_0.6:_ hexagonal tubular carbon nitride possesses abundant nitrogen defects and oxygen‐containing defects through the H3PO4‐assisted hydrothermal method;

^g)^ D‐TCN_450_: tubular carbon nitride with defects;

^h)^ SD‐CN: the modified carbon nitride with sodium doping and nitrogen defect;

^i)^ g‐C_3_N_4_‐U: g‐C_3_N_4_ synthesized from urea;

^j)^ bmw‐DCN‐x: defective carbon nitride with a mass ratio of sodium chloride to dicyandiamide of 30:1 in the ball‐milled samples;

^k)^ U/AC_0.5_: porous g‐C_3_N_4_ aerosols U/AC_0.5_ (0.5 represents the molar ratio of azodicarbonamide or azoformamide (AC) to urea);

^l)^ A‐V‐g‐C_3_N_4_: atomic‐layered g‐C_3_N_4_ with nitrogen vacancy;

^m)^ CNQ680: graphitic carbon nitride with quick (5 min) thermal treatment method at 680 °C;

^n)^ DCN‐m: defective carbon nitride by mixing BCN, LiCl, and KCl;

^o)^ TCNS: defective carbon nitride modified with S and cyano groups, obtained through a two‐step calcination process in a tubular furnace;

^p)^ MTCN: defective carbon nitride synthesized from melamine and thiourea;

^q)^ SCN‐x: 3D sponge‐like porous carbon nitride;

^r)^ CACN: crystalline‐amorphous carbon nitride.

### 2.1 Synthetic Strategies and Function Mechanism of Vacancies

Commonly, the first category that should be mentioned is vacancy generation, in which nitrogen‐vacancies and carbon‐vacancies are both compelling.^[^ ^73^, ^74^, ^77^, ^78^ ^]^ The formation of these vacancies might promote charge carrier separation and strengthen surface molecule adsorption. Additionally, the existence of vacancies is vital when considering the introduction of element doping and functional groups on the structure of carbon nitride, which has an effect on the capacity of the surrounding atoms of vacancies.

**Figure 2:**
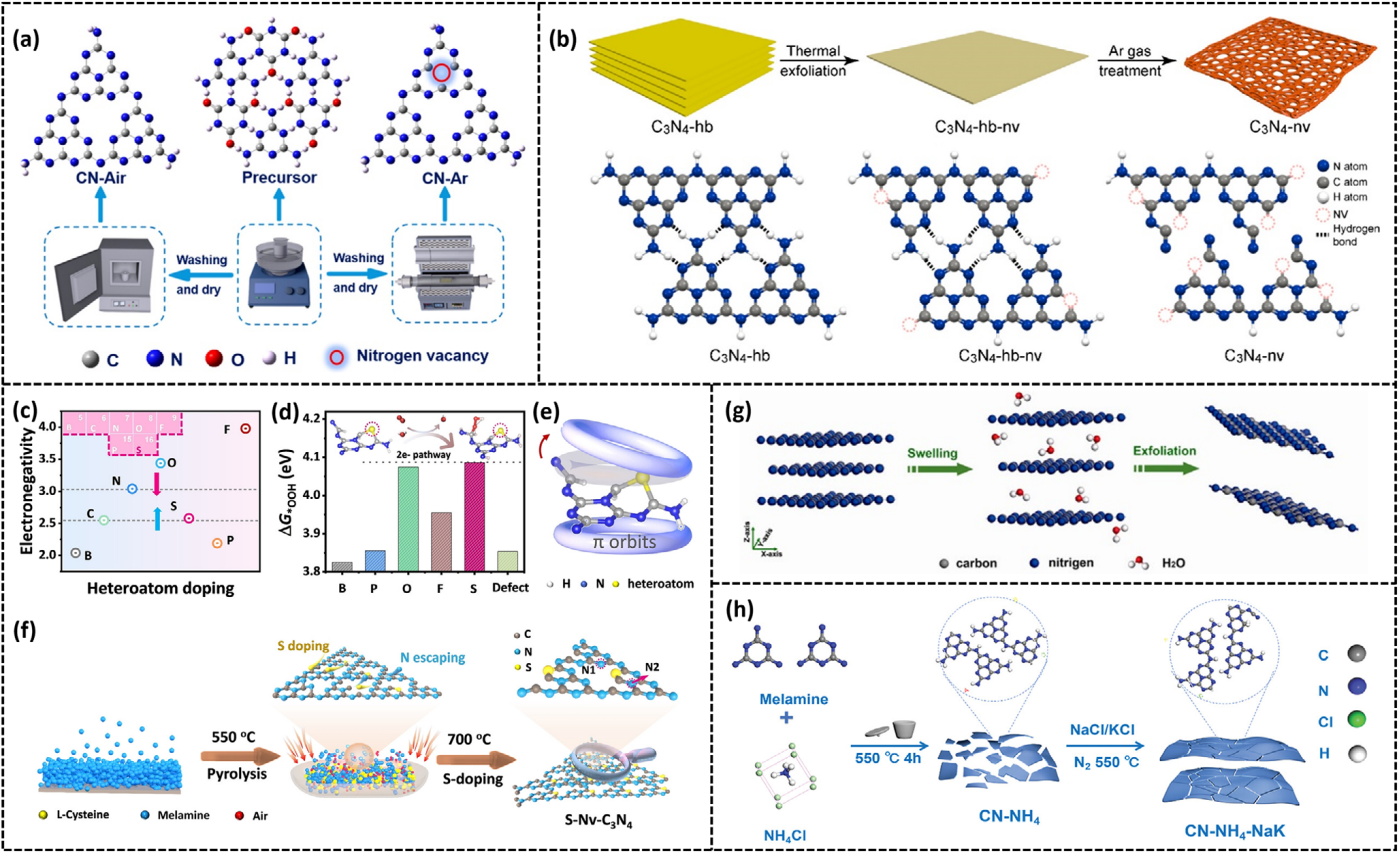
a) Preparation for CN‐Air and CN‐Ar. Reproduced with permission.^[^ ^73^ ^]^ Copyright 2023, American Chemical Society. b) Synthetic process and structure of C_3_N_4_‐hb, C_3_N_4_‐hb‐nv, and C_3_N_4_‐nv. Reproduced with permission.^[^ ^74^ ^]^ Copyright 2024, American Chemical Society. c) Electronegativity of different non‐metals, d) adsorption energy of •OOH of different structures, e) schematic diagram of the electronic localization mechanism, f) synthetic route for S‐Nv‐C_3_N_4_. Reproduced with permission.^[^ ^75^ ^]^ Copyright 2024, Wiley‐VCH. g) Liquid‐exfoliation process from bulk g‐C_3_N_4_ to ultrathin nanosheets. Reproduced with permission.^[^ ^76^ ^]^ Copyright 2012, American Chemical Society. h) Synthesis process for CN‐NH_4_‐NaK. Reproduced with permission.^[^ ^43^ ^]^ Copyright 2024, Wiley‐VCH.

Taking N‐vacancy as an example, Wu's group^[^ ^73^ ^]^ prepared nitrogen‐vacancy carbon nitride with N_3C_ vacancies through the synergistic action of argon pyrolysis and the self‐assembly of melamine and cyanuric acid in one step (**Figure** **2**a), in which Ar thermal procedure is essential for the etching process and defects introduction in structural units, thus further weakening the interlayer stacking and increased active sites. Self‐assembled precursors might produce active free radical fragments during the calcination in an inert atmosphere (Ar), and these active radicals may attack the N site for the formation of N vacancies, thus promoting the adsorption of H^+^ and oxygen (O_2_) and accelerating charge separation. In another example of N‐vacancy, a post‐thermal treatment under the inert atmosphere was utilized for the formation of nitrogen vacancies and cyano groups inside carbon nitride by Wang's group^[^ ^74^ ^]^ (Figure 2b), thus promoting surface adsorption and O_2_ reduction through the formed hydrogen bond, while the exposed N atoms could perform as the proton buffering sites and accelerate H_2_O_2_ generation. This study carried out the importance of hydrogen bond construction between the photocatalyst and reactants, and listed the importance of vacancies‐modified carbon nitride.

Additionally, carbon vacancies have been investigated for promoting the performance of carbon nitride, which is beneficial for the bandgap alignment for optical adsorption ability and further distribution of dopants. For instance, the introduction of carbon vacancies has been investigated for H_2_O_2_ photosynthesis by Wang's group^[^ ^52^ ^]^ with a 14 times enhancement, in which carbon vacancies might reduce the symmetry of g‐C_3_N_4_ and effect electron delocalization. Moreover, the narrowed bandgap, enhanced light absorption, and the high selectivity for a one‐step two‐electron direct reduction pathway are probed in this interesting research work. Focalizing attention in the influences of vacancies, it is well acknowledged that vacancy defects are able to trap photo‐generated electrons, thus suppressing charge recombination.^[^ ^79^, ^80^ ^]^ Besides, vacancies have been confirmed to promote surface adsorption capacity and accelerate the activation of reactants owing to the abundant localized electrons.^[^ ^81^ ^]^ Briefly, having a basic idea of the main principles for vacancy‐engineering procedure helps to provide valuable guidance for the modification of carbon nitride toward efficient light harvesting.

### 2.2 Formation and Function Mechanism of Dopants and Functional Groups

To manipulate the electronic and band structure of carbon nitride, atomic dopants and functional groups introduction have been extensively investigated to further enhance the photocatalytic H_2_O_2_ generation performance.^[^ ^82^, ^83^, ^84^, ^85^, ^86^, ^87^ ^]^ Generally speaking, the performance of g‐C_3_N_4_ is usually restricted by the latter process due to the difficulties in the formation of •OOH and subsequent hydrogenation, which can be attributed to the surface adsorption and desorption capacities. Inspired by this situation, Wen et al.^[^ ^88^ ^]^ prepared a protonation‐induced oxygen doping over g‐C_3_N_4_ through a co‐calcination of urea with oxalic acid, followed with the dispersion in hydrochloric acid solution for protonation, thus constructing irregular nanosheet‐like structures with a large surface area. Intriguingly, it has been confirmed that the introduction of oxygen dopants not only have replaced the position of nitrogen atoms in the structure, but also might be beneficial for the exfoliation of bulk carbon nitride into a smaller size, which is vital for the further oxygen adsorption and H_2_O_2_ desorption.

In another meaningful study, based on the theoretical demonstration of electronic localization mechanism over varying atoms doping including C, N, B, P, F, O and S, a novel strategy was proposed by Zhang's group^[^ ^75^ ^]^ to promote the localized electron polarization for enhance the ferromagnetism of ultra‐thin 2D carbon nitride nanosheets (Figure 2c–e). Thereinto, a typical sulfur‐doped defect‐rich graphitic carbon nitride (S‐Nv‐C_3_N_4_) was synthesized by through facile one‐pot segmented polymerization in a completely enclosed crucible (Figure 2f), accompanied with the dehydration of L(+)‐cysteine, the formation of N─H bonds, and the production of pyrrole‐N. Of note, S doping can promote localized ferromagnetic coupling, induce long‐range ferromagnetic ordering, promote electron enrichment, accelerate charge transfer, and strengthen enhances the •OOH desorption. Such a research provides an in‐deep investigation and vital guidance when designing element‐doping carbon nitride, with the consideration of in‐plane π‐electron rearrangement and electrons distribution.

Another typical category of vacancies is functional groups, which is advantageous for the electrons migration and surface reactant adsorption.^[^ ^89^ ^]^ For example, a carbon nitride framework with a sodium cyanaminate moiety was developed, and its photocatalytic performance was investigated by Choi's group.^[^ ^90^ ^]^ For brevity, the introduction of such functional groups can promote photon absorption and alter the energy landscape of the structure, thus accelerating surface oxygen adsorption and accelerating charge transportation. In another recent case, Zou and his co‐workers^[^ ^91^ ^]^ found that N‐hydroxymethyls groups (─NH─CH_2_‐OH) introduced onto g‐C_3_N_4_ is of vital importance for the enhancement of water dehydrogenation, oxygen adsorption, and the reduction kinetics of oxygen, while some characterizations including light absorption and band structures are not influenced in this situation, providing a meaningful reference for modified g‐C_3_N_4_ for the kinetic perspective. Sparked by this inspiration, a vital factor should be considered for superior photocatalytic H_2_O_2_ generation performance the surface reactants adsorption and products desorption capacity, which is a basic design principle for ideal photocatalytic systems.

### 2.3 Synthesis and Function of Structural Disorder Engineering

Considering the advantages of ultrathin 2D nanosheets for abundant active sites formation and high light utilization efficiency, countless investigations have been explored over the structure modification of carbon nitride, with exceptional electronic and chemical‐physical properties. For instance, Xie's group^[^ ^76^ ^]^ synthesized ultrathin graphitic‐phase C_3_N_4_ nanosheets through a “green” liquid exfoliation route from bulk g‐C_3_N_4_ in water for the first time in 2012, synthesizing a stable photocatalyst in both the acidic and alkaline environments (Figure 2g). As demonstrated, single‐layered g‐C_3_N_4_ nanosheets show an increase of DOS at the conduction band edge with respect to the bulk counterpart, indicating it possessed more carriers. Inspired by this, defect‐engineering like structural disorder construction, are conducted when modulating the structure and morphology of carbon nitride.^[^ ^92^, ^93^, ^94^ ^]^

Taking an example, Cheng et al.^[^ ^95^ ^]^ prepared a disordered nitrogen‐defect‐rich porous GCN long‐range atomic disordered structure for photocatalytic reactions, with rich nitrogen defects in the in‐planes of disordered carbon nitride networks, displaying extended light absorption and available reaction active sites for efficient charge separation. Interestingly, disordered samples with long‐range disorder and short‐range order might lead to modulated electronic, optical, and mechanical properties. In another typical research, Wang's group^[^ ^43^ ^]^ tried to fragment carbon nitride into smaller pieces and construct multiple order–disorder interfaces in the as‐synthesized CN‐NH_4_‐NaK (Figure 2h), thus promoting charge dynamics and generating more separated redox centers, and providing a design pathway for constructing a homojunction structure with multiple interface configurations in carbon nitride networks, which might be advantageous for the improvement of solar‐to‐chemical energy conversion.

In summary, structural disorder engineering, through the introduction of controllable defects and the reconstruction of ordered–disordered interfaces, offers an innovative strategy for optimizing the photocatalytic performance of carbon nitride.^[^ ^96^ ^]^ The core advantages of this design principle are reflected in 3D: a) The construction of in‐plane nitrogen defects effectively modulates the distribution of electronic states density, enhancing the kinetics of carrier migration; b) The composite structure of long‐range disorder and short‐range order broadens the light response range through localized reconstruction of electronic states; c) Multiscale interface engineering creates abundant heterojunction active sites, achieving spatial decoupling and separation of redox reaction centers. It is worth noting that the construction of such non‐equilibrium structures requires precise control of the balance between thermodynamic driving forces and kinetic limitations.

## 3 Recent Progress Concerning Band Structure Engineering, Electronic Structure Analysis, and Pathway Regulation

Recent researches concerning carbon nitride‐based materials through defect engineering have shown its capacities for efficient solar‐to‐chemical conversion, with broadened light adsorption, modulated band structures, adjustable active sites, increased surface area, promoted oxygen adsorption ability, and strengthened charge transfer and separation (**Table** **2**). Furthermore, the pathway selectivity for H_2_O_2_ photosynthesis was also discussed clearly in diverse systems, demonstrating the factors influencing the whole redox reaction. In this section, various representative parts as well as the strengths and limitations of these synthetic strategies are discussed in detail.

**Table 2:** Performances of defective g‐C_3_N_4_ for H_2_O_2_ photosynthesis.

Photocatalysts	type of defect	Light source	Sacrificial agent	H_2_O_2_ generation	Refs.
Cv‐g‐C_3_N_4_	C defects	λ> 420 nm	deionized water	96 µm h^−1^ (100 mg)	[52]
CNH	C defects	320 nm‐780 nm	10% EtOH	45 µm h^−1^ (100 mg)	[53]
IO CN‐Cv^a)^	C defects	λ> 420 nm	5% EtOH	162.87 µm h^−1^ (20 mg)	[97]
MACN^b)^	C defects	λ> 420 nm	10% isopropanol	411 µm h^−1^ (5 mg)	[98]
UCNS_580_ ^c)^	C vacancy and C ≡ N group	λ> 420 nm	20% EtOH	2083.5 µm h^−1^ (500 mg)	[99]
A_0.05_CN^d)^	C doping and ─OH modification	λ> 420 nm	10% EtOH	1800 µm h^−1^ (5 mg)	[100]
3D‐CN‐C1^e)^	C defects	λ> 400 nm	5% isopropanol	125.75 µm h^−1^ (20 mg)	[101]
g‐C_3_N_4_‐N_3C_‐0.3	N defects	AM 1.5	10% methanol	109.8 µm h^−1^ (10 mg)	[56]
C_3_N_4_‐nv	N defects	λ> 420 nm	deionized water	75.66 µm h^−1^ (50 mg)	[74]
DPCN‐520^f)^	N defects	λ> 420 nm	10% EtOH	283 µm h^−1^ (100 mg)	[102]
NVCNS	N defects	300 W Xe lamp	10% isopropanol	2206.55 µm h^−1^ (25 mg)	[103]
CNK_0.2 _ ^g)^	N defects	λ> 420 nm	5% methanol	1010 µm h^−1^ (0.1 g)	[80]
R_370_‐CN^h)^	N defects	λ> 420 nm	pure water	170 µm h^−1^ (0.1 g)	[104]
CKCN‐0.03^i)^	N defects	λ> 420 nm	20% EtOH	1907.5 µm h^−1^ (20 mg)	[57]
PCN‐NV_C_	N defects	λ> 420 nm	10% methanol	836.7 µm h^−1^ (50 mg)	[105]
Nv‐CNN‐3	N defects	λ> 420 nm	10% EtOH	884 µm h^−1^ (25 mg)	[106]
g‐C_3_N_4_‐0.05	N defects and KOH‐doping	λ> 420 nm	10% isopropanol	675.84 µm h^−1^ (30 mg)	[107]
Nv/Cyano‐PCN0.4	N defects and ─C ≡ N	λ> 420 nm	5% EtOH	3930.9 µm h^−1^ (50 mg)	[108]
DDCN^j)^	N defects and ─C ≡ N	λ> 420 nm	10% methanol	206.2 µm h^−1^ (20 mg)	[109]
B_3_CN^k)^	N defects and ─C ≡ N	λ> 420 nm	deionized water	114 µm h^−1^ (10 mg)	[110]
2%*Ox*‐KPHI^l)^	N defects and ─C ≡ N	410 nm LED	2 mL of 10% w/w glycerin	10 mm h^−1^ (5 mg)	[111]
CN‐U20M^m)^	N defects and ─C ≡ N	λ> 420 nm	10% EtOH	4376 µm h^−1^ (20 mg)	[112]
Bi_3.6_K_3_‐CN	K doping and B doping	λ> 420 nm	10% EtOH	402.57 µm h^−1^ (30 mg)	[113]
K‐CN	K doping and N defects	λ> 420 nm	10% isopropanol	19 663 µm h^−1^ (50 mg)	[114]
ACNN	alkali metal dopants and N defects	λ> 420 nm	10% isopropanol	102 mm h^−1^ (25 mg)	[115]
NCN‐2AP‐10^n)^	pyridine rings and N defects	λ> 420 nm	10% isopropanol	193.68 µm h^−1^ (20 mg)	[2]
OCN8^o)^	oxygen‐rich and N defects	λ> 420 nm	5 mmol L^−1^ Na_3_PO_4_ and 10% isopropanol	5781 µm h^−1^ (60 mg)	[116]
ASCN‐3^p)^	carbonyl modification and N defects	AM 1.5	40% acetonitrile and 0.2 mmol 4‐methoxybenzyl alcohol	4040 µm h^−1^ (10 mg)	[117]
CNNK	K/Na dopants and N defects	λ> 420 nm	10% EtOH	6289.6 µm h^−1^ (50 mg)	[118]
CN‐O‐AQ^q)^	N/O dopants and cyanonitrogen defects	λ> 400 nm	50% EtOH	78.31 mm h^−1^ (10 mg)	[119]
BPMC‐Vs^r)^	B/P dopants, N defects and ─C ≡ N	λ> 420 nm	deionized water	85.3 µm h^−1^ (30 mg)	[120]
BCN‐L^s)^	B dopant and N defects	λ> 420 nm	10% isopropanol	331.4 µm h^−1^ (20 mg)	[121]
KCN‐C	C, K co‐doping and N defects	AM 1.5	10% isopropanol	2708 µm h^−1^ (25 mg)	[122]
KLCN^t)^	ring‐embedded and N defects	λ> 420 nm	10% isopropanol	1856.79 µm h^−1^ (50 mg)	[123]
Ag@MCT^u)^	Ag single atoms and N defects	λ> 420 nm	deionized water	151 µm h^−1^ (20 mg)	[124]
NaSCN	Na dopant, ─C ≡ N and N defects	320 to 780 nm	10% EtOH	109.55 µm h^−1^ (100 mg)	[125]
KPCN	K/P dopants and ─C ≡ N	λ> 420 nm	10% isopropanol	387 µm h^−1^ (50 mg)	[126]
O/K‐PCN	O/K dopants and ─C ≡ N	λ> 420 nm	5% EtOH	2560 µm h^−1^ (50 mg)	[127]
CN‐K_4_Na_2_	heptazine/triazine junction and cyanamide defect	λ> 400 nm	25% EtOH	15 mm h^−1^ (5 mg)	[128]
CACN	order–disorder interfaces	AM 1.5	10% EtOH	638.3 µm h^−1^ (20 mg)	[16]

^a)^ IO CN‐Cv: carbon nitride with inverse opal (IO) structure and carbon vacancies;

^b)^ MACN: the carbon nitride nanowire clusters synthesized in an argon atmosphere;

^c)^ UCNS_580_: ultrathin carbon nitride formed by calcination at 580 °C;

^d)^ A_0.05_CN: carbon nitride synthesized from a mixture with a molar ratio of acetylacetone to urea of 0.05;

^e)^ 3D‐CN‐C1: carbon‐deficient 3D hierarchical porous C_3_N_4_;

^f)^ DPCN‐520: carbon nitride with nitrogen vacancies calcined at 520 °C;

^g)^ CNK_0.2_: 0.2 g KOH modified graphitic carbon nitrides;

^h)^ R_370_‐CN: the reduction product of g‐C_3_N_4_‐450 at 370 °C;

^i)^ CKCN‐0.03: calcination product of 0.03 g KOH with CN;

^j)^ DDCN: dual‐deficient CN;

^k)^ B_3_CN: The sample obtained by calcination at a mass ratio of NaBH_4_ to CN of 3;

^l)^ 2%Ox‐KPHI: defective carbon nitride prepared from 2% oxamide;

^m)^ CN‐U20M: defective carbon nitride prepared with a urea/melamine ratio of 20;

^n)^ NCN‐2AP‐10: nitrogen‐deficient carbon nitride containing 10 mg of 2‐aminopyridine;

^o)^ OCN8: oxygen‐rich g‐C_3_N_4_ with abundant nitrogen vacancies after 8 h of stirring;

^p)^ ASCN‐3: carbon nitride with thin layers assemble;

^q)^ CN‐O‐AQ: the sample obtained by covalent bonding of g‐C_3_N_4_ with anthraquinone through ester C─O─C═O oxygen bridges;

^r)^ BPMC‐Vs: carbon nitride with multiple heteroatoms (B and P) and multiple defects;

^s)^ BCN‐L: laser‐modified graphitic carbon nitride;

^t)^ KLCN: lignin‐derived carbon ring‐embedded and nitrogen defect co‐doped g‐C_3_N_4_;

^u)^ Ag@MCT: Ag single atoms and nitrogen defects decorated carbon nitride.

### 3.1 Band Structure Engineering for H_2_O_2_ Generation

**Figure 3:**
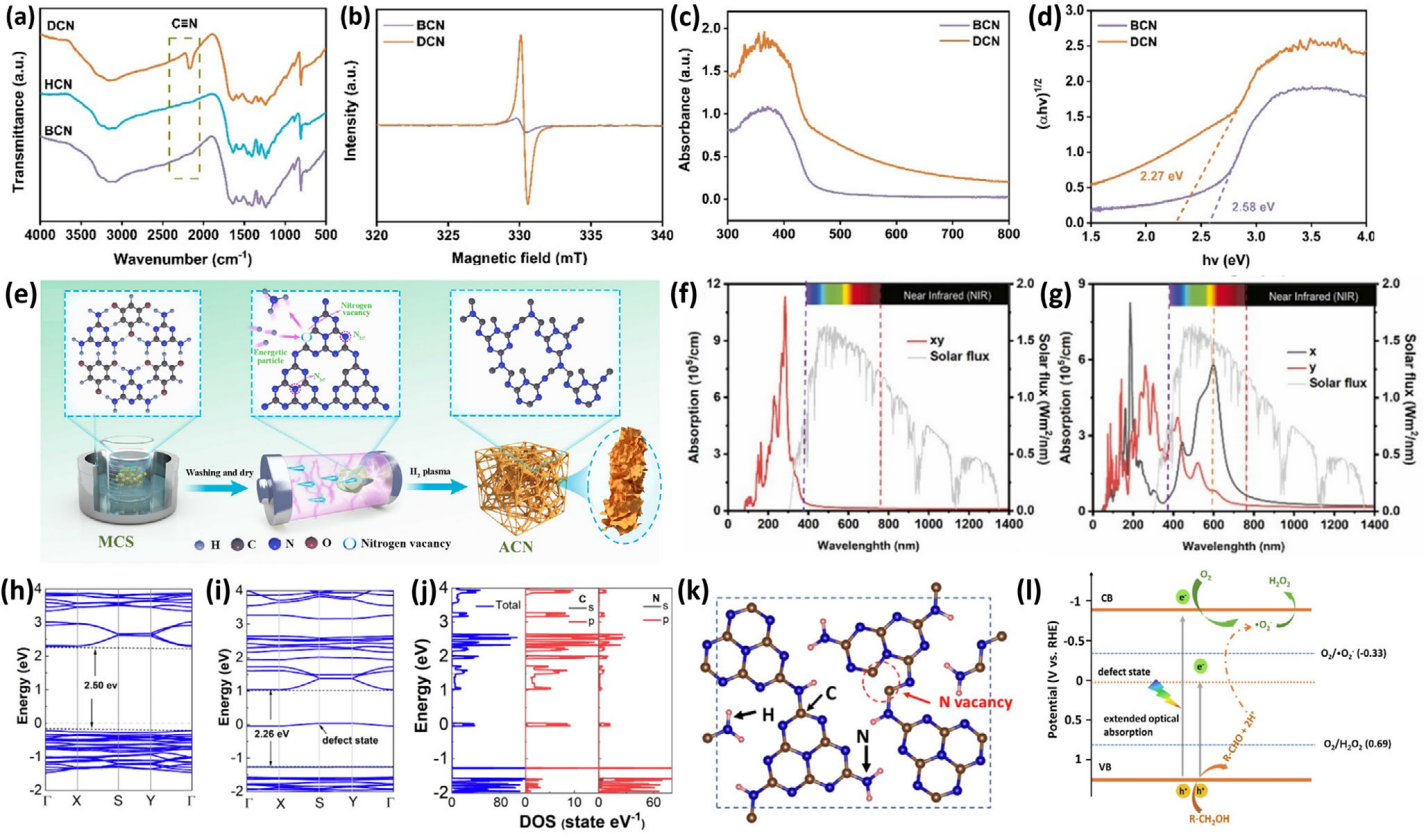
a) FTIR spectra of DCN, HCN, and BCN, b) EPR spectra, c) UV–vis absorption spectra, and d) bandgap of BCN and DCN. Reproduced with permission.^[^ ^131^ ^]^ Copyright 2024, Elsevier. e) Schematic synthesis of ACN, f,g) optical absorption along the x and y directions of BCN and CAN. Reproduced with permission.^[^ ^132^ ^]^ Copyright 2022, Elsevier. h,i) The band structures for BCN and NVCNS. j–l) DOS, structure models, and band structure of NVCNS. Reproduced with permission.^[^ ^103^ ^]^ Copyright 2021, Elsevier.

Overall, defect engineering plays a crucial role in modulating the band structure of carbon nitride, including bandgap,^[^ ^20^, ^104^ ^]^ conduction band (CB) position,^[^ ^80^ ^]^ and valance band (VB) position,^[^ ^129^ ^]^ which not only influences the optical absorption range,^[^ ^130^ ^]^ but also might be a factor for valid charge separation.^[^ ^39^, ^50^ ^]^ Taking an example, Sun et al.^[^ ^131^ ^]^ synthesized carbon nitride materials using the hydrothermal method and then introduced nitrogen vacancies by simple calcination with NaBH_4_, and the presence of nitrogen vacancies was confirmed using Fourier transform infrared (FTIR) spectra and electron paramagnetic resonance (EPR) spectra (**Figure** **3**a,b). Intriguingly, it was observed that a more intense C ≡ N asymmetric stretching vibration peak appeared for defective g‐C_3_N_4_ (DCN) as compared to hydrochloric g‐C_3_N_4_ (HCN) and bulk g‐C_3_N_4_ (BCN), and the heptazine units in carbon nitride were destructed though the import of the nitrogen vacancies, introducing more unsaturated sites, which contributed to the significantly enhanced EPR signal intensity of DCN. Following this, the UV–vis absorption spectra and bandgap energy diagram derived from the UV–vis absorption spectra are exhibited in Figure 3c,d, indicating the broadening of the optical absorption range and decreased bandgap of DCN after the introduction of nitrogen defects. This research describes the influences of nitrogen defects on the modulation of material structure and bandgap, which influence the light absorption and extend the absorption edge, which is beneficial for promoting artificial solar‐to‐chemical conversion in practical applications.

Additionally, in another interesting research work,^[^ ^132^ ^]^ the absorption range was broadened to over 593 nm, which is advantageous for more efficient utilization of solar energy. In this case, a one‐step plasma method was applied to dissolve melamine cyanuric acid in sulfuric acid and was further treated in a tubular furnace with H_2_ plasma. It is noteworthy that H_2_ could act as high‐energy particles attacking the N lattice sites for the formation of carbon nitride with N_2C_ vacancies (Figure 3e), with apparent enhanced light adsorption and a narrowed bandgap of 2.24 eV for amorphous carbon nitride (ACN) as compared to BCN. To provide a more detailed explanation of the enhancement in light absorption, photo absorption experiments were carried out (Figure 3f,g). Delightedly, it is evident that ACN has a stronger capability for sunlight absorption in the visible and near‐infrared regions, which can be attributed to defect engineering is the asymmetric structure leading to anisotropic optical absorption along the *x*‐ and *y*‐directions. In another novel study, N‐vacancy graphitic carbon nitride spheres (NVCNS) were prepared using a low‐temperature heating method in H_2_ plasma by Huang's group.^[^ ^103^ ^]^ In this work, based on the experimental broadened light absorption range and a reduced bandgap, first‐principles calculations were applied to demonstrate the band structure and density of states for BCN and NVCNS (Figure 3h–j). On the basis of broadly similar band structures, new defect energy states emerged in NVCNS. After the introduction of N vacancies, the bandgap decreased from 2.50 to 2.26 eV, which is consistent with the results from UV–vis DRS and the bandgap energy diagram (Figure 3h,i). As displayed in Figure 3j, the defect energy bands of NVCNS are composed of N *2p* and C *2p* orbitals, and the valence band maximum and conduction band minimum of NVCNS are also closely related to the C *2p* and N *2p* orbitals. Meanwhile, the molecular structure and band structure of NVCNS were shown in Figure 3k,l, defect states were introduced into the band structure of NVCNS due to the existence of defects, thus reducing the band width for electron transition and accelerating charge transfer for H_2_O_2_ photosynthesis. The incorporation of vacancies into the carbon nitride has been confirmed to broaden the visible light absorption for strengthened utilization of solar light, which is consistent with the environmentally friendly and energy‐saving prospect of the society development.

**Figure 4:**
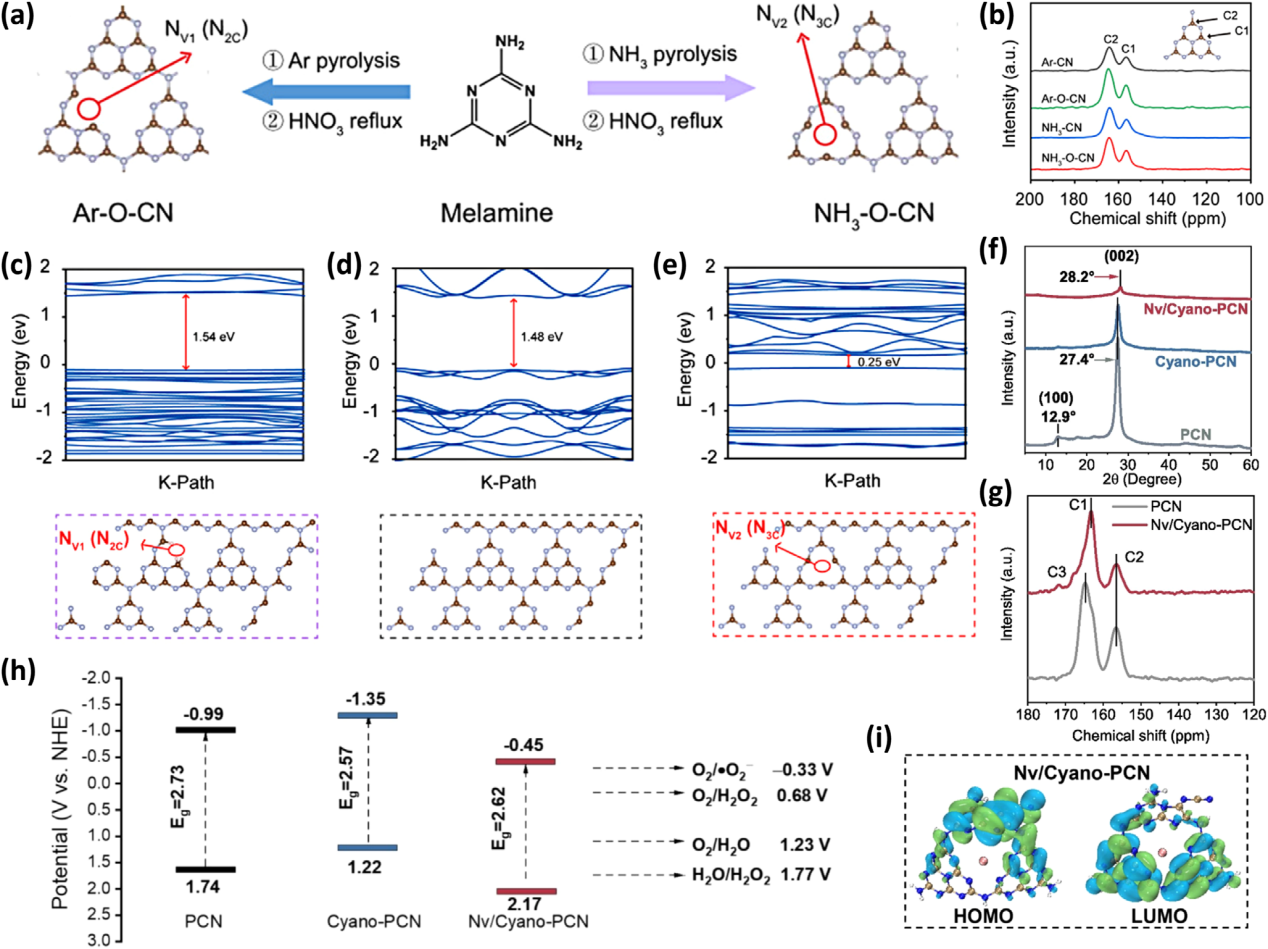
a) Schematic synthesis of Ar─O─CN and NH_3_─O─CN, b) the solid‐state ^13^C NMR spectra of Ar─CN, Ar─O─CN, NH_3_─CN and NH_3_─O─CN, c–e) the theoretically calculated band structure and structure model of Ar─O─CN, Ar─CN, and NH_3_─O─CN. Reproduced with permission.^[^ ^133^ ^]^ Copyright 2022, Wiley‐VCH. f) XRD patterns, g) the solid‐state ^13^C NMR spectra, h) band structures analysis, i) HOMO and LUMO distribution of Nv/Cyano‐PCN. Reproduced with permission.^[^ ^108^ ^]^ Copyright 2024, Elsevier.

For a valid photocatalyst, the band positions are also crucial in addition to the bandgap, which is pregnant for the motivated oxygen reduction or water oxidation reaction for H_2_O_2_ formation. In an inspiring work, different types of nitrogen vacancies (N_2C_ and N_3C_) were formed by introducing different pyrolysis atmospheres, yet their structure‐led band positions also varied.^[^ ^133^ ^]^ As shown in **Figure** **4**a, melamine pyrolyzed under an Ar atmosphere and refluxed in nitric acid forms N_2C_ vacancies, while the gas flow is changed to NH_3_, N_3C_ vacancies are formed. Subsequently, the presence of N_2C_ and N_3C_ was confirmed by solid‐state ^13^C nuclear magnetic resonance (NMR) spectra (Figure 4b), and the existence of vacancies was proven by XPS spectra and EPR spectra. The theoretical calculations of the band structures for Ar‐atmosphere‐derived carbon nitride (Ar─CN), Ar‐synthesized and acid‐treated carbon nitride (Ar─O─CN), and NH_3_‐synthesized and acid‐treated carbon nitride (NH_3_─O─CN) are displayed in Figure 4c–e, in which NH_3_─O─CN showed the smallest calculated band gap and a more positive CB, indicating that electrons theoretically can more easily transition from the VB to CB. In other experimental validations, it was indeed confirmed that NH_3_─O─CN with a more positive CB more readily enjoys photocatalytic activity. This study has described the impact of different types of nitrogen defects on the band structure and photocatalytic activity, which is beneficial for the targeted and efficient introduction of specific types of nitrogen defects.

Differently, the dual‐defective carbon nitride photocatalyst, including both cyano and nitrogen vacancies, was obtained by Huo's group^[^ ^108^ ^]^ through a salt‐assisted post‐treatment strategy, and this study demonstrated the dramatic impact of defect engineering on the electronic orbits of the material in a computational manner. Preferably, the planar structure of carbon nitride is disrupted owing to the introduction of defects, leading to the disappearance of the peak corresponding to the 100 facet of nitrogen and cyanide vacancies‐enriched carbon nitride (Nv/Cyano‐PCN) material in the XRD pattern (Figure 4f). On the other hand, the disruption of the interlayer structure in Nv/Cyano‐PCN might lead to the shift of the peak corresponding to the 200 facet toward a larger angle. Second, a new peak was observed in the solid‐state ^13^C NMR spectrum, which corresponds to the carbon signal of N─C ≡ N, representing a tangible manifestation of cyano defects (Figure 4g). Based on experimental results, the band structures of the catalyst have been determined, as displayed in Figure 4h. The VB and CB positions of Nv/Cyano‐PCN are more positive compared to those of cyanide vacancie‐enriched carbon nitride (Cyano‐PCN) and PCN. Ultimately, DFT calculations were employed to illustrate the bonding and antibonding orbitals of Nv/Cyano‐PCN (Figure 4i). Contrary to the uniform distribution of HOMO and LUMO of the sample, the electrons in HOMO of Nv/Cyano‐PCN are predominantly localized around the dual‐defect groups, while the electrons in LUMO of Nv/Cyano‐PCN are inversely concentrated in the defect‐free areas, making the internal processes more efficient and naturally enhancing the production efficiency of H_2_O_2_.

The notion that carbon nitride with vacancies possesses an intrinsic band structure more suitable for H_2_O_2_ production has been accepted as an acknowledged perspective, offering a desirable approach for the design of efficient solar‐energy‐conversion photocatalytic systems. Considering that defect structures can modulate the defect levels of carbon nitride, alter the band positions, and change the range of light absorption, these changes indeed have a significant impact on the yield of H_2_O_2_. Therefore, by rationally designing and regulating defect structures, the performance of carbon nitride in photocatalytic H_2_O_2_ production can be considerably enhanced, offering an effective solution for the development of highly efficient photocatalytic materials.

### 3.2 Tuning Electronic Structure for H_2_O_2_ Photosynthesis

**Figure 5:**
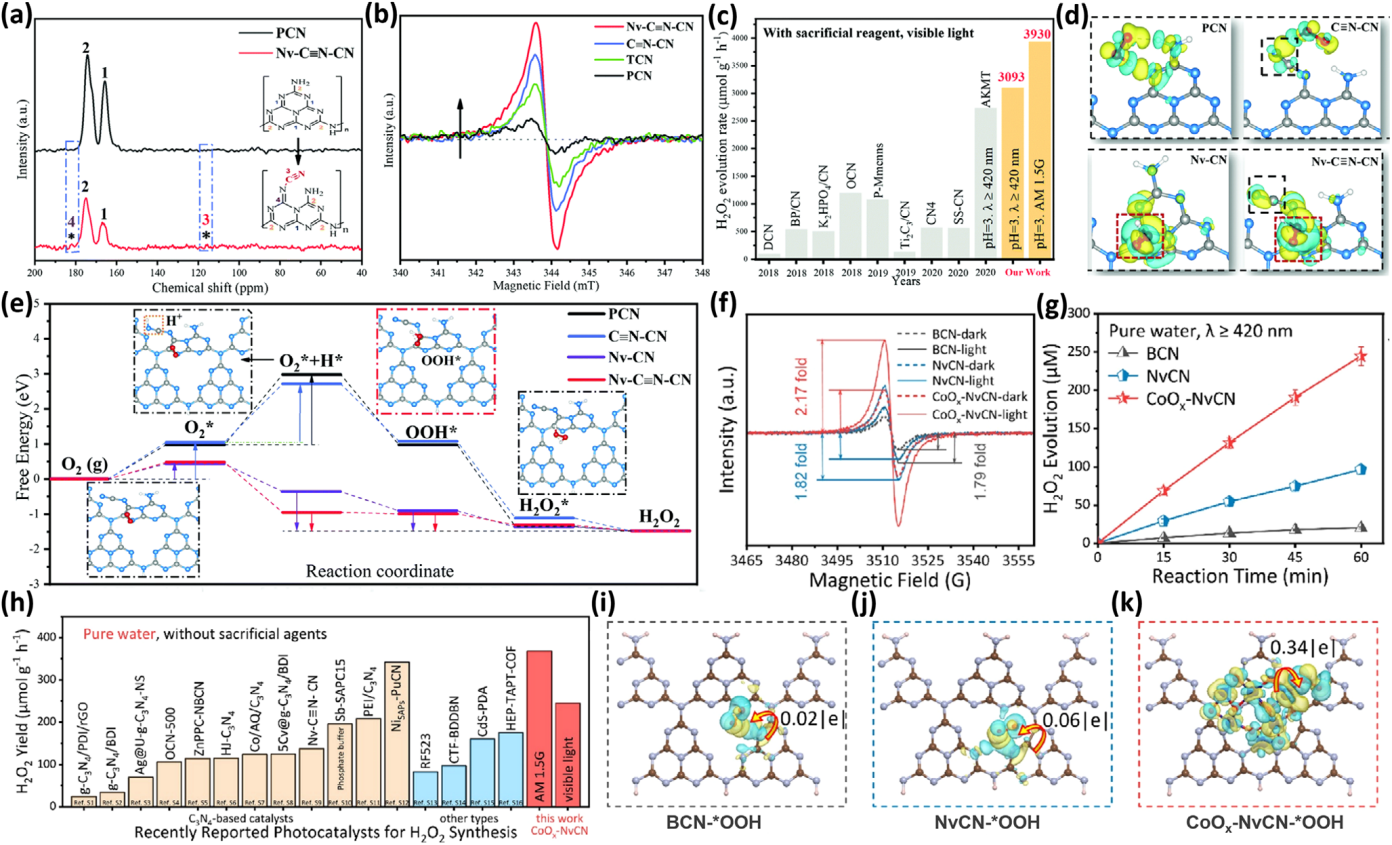
a) The solid‐state ^13^C NMR spectra of PCN and Nv─C ≡ N─CN, b) EPR spectra of samples, c) comparison of the H_2_O_2_ generation, d) charge density differences (isosurface value 0.0025 eV Å^−3^) between •OOH group and PCN, C ≡ N─CN, Nv─CN, and Nv─C ≡ N─CN. e) Free energy profiles for photocatalytic H_2_O_2_ evolution. Reproduced with permission.^[^ ^28^ ^]^ Copyright 2022, RSC. f) The EPR spectra and g) H_2_O_2_ evolution of BCN, NvCN, and CoO_x_‐NvCN, h) comparison of CoO_x_‐NvCN with recently reported photocatalysts under the standardized H_2_O_2_ yield (µmol g^−1^ h^−1^), i–k) charge density differences (isosurface value 1 × 10^−3^ |e|  bohr^−3^) between •OOH group and BCN, NvCN, and CoO_x_‐NvCN. Reproduced with permission.^[^ ^134^ ^]^ Copyright 2024, American Chemical Society.

Additionally, the role of defect engineering in the adsorption capacity of photocatalysts can be reflected in the modulation of electronic structure and the regulation of active sites, which is noteworthy for strengthened surface reactant adsorption and charge separation. Taking an example, a process involving thermal polymerization with thiourea and subsequent heat treatment in Ar was developed by Zhang et al.^[^ ^28^ ^]^ to introduce dual defects, including ─C ≡ N groups and nitrogen vacancies into carbon nitride. The advent of novel peaks (designated as 3 and 4) as depicted in the solid‐state ^13^C NMR spectra (**Figure** **5**a) successfully demonstrated the synthesis of the photocatalyst, in which peak 3 is ascribed to the cyano carbon and peak 4 represents the carbon atom in proximity to the cyano group. Besides, the remarkable intensity of the EPR signal observed for Nv─C ≡ N─CN in Figure 5b is a consequence of the robust electron‐withdrawing properties of the ─C ≡ N groups, coupled with the electron‐deficient characteristics of nitrogen vacancies. These features, in turn, exert a significant influence on the electronic structure of Nv─C ≡ N─CN, thereby not only enhancing its electronic properties but also serving as a definitive affirmation of the successful implementation of defect engineering techniques. Intriguingly, Nv─C ≡ N─CN demonstrated an exceptional activity under visible light at pH 3, and the H_2_O_2_ yield has soared to an impressive 3093 µm h^−1^, outperforming some other catalytic systems (Figure 5c). It is well acknowledged that the hydroperoxyl radical (•OOH) can serve as a pivotal intermediate in the genesis of H_2_O_2_. Notably, the pronounced charge redistribution observed between Nv─C ≡ N─CN and •OOH surpasses that of the interactions between PCN, C ≡ N─CN, and Nv─CN, underscoring the significant enhancement in the active sites density owing to the strategic implementation of defect engineering (Figure 5d). This enhancement suggests a more vigorous and efficient interaction with the key intermediate, highlighting the transformative impact of defect engineering on the material's photocatalytic capabilities. The charge difference results align with the free energy profile of the 2e^−^ ORR pathway, where the photocatalyst with introduced defects exhibits lower energy requirements at each stage (Figure 5e). The incorporation of reactive active sites renders the entire energy profile more stable. Specifically, the transition from [O_2_^*^+H^*^] to [•OOH] only demands 0.04 Gibbs free energy, which aptly elucidates the superior photocatalytic performance of Nv─C ≡ N─CN. This study elucidates the key role of dual defect sites in photocatalytic surface reactions, where N vacancies can effectively adsorb and activate O_2_, while ─C ≡ N groups enhance the adsorption of H^+^, synergistically promoting the conversion of the key intermediate •OOH to H_2_O_2_. More importantly, DFT calculation was employed to conduct an in‐depth analysis of the electronic structure of Nv─C ≡ N─CN, and EPR spectroscopy was combined to show the impact of defects on the electronic structure. In fact, a unique electron‐rich structure was constructed through the introduction of dual defects of N vacancies and ─C ≡ N groups, thus accelerating the adsorption of O_2_ and H^+^. Such an interesting research systematically analyzes the impact of dual defect sites on the photocatalytic transformation process, with charge density distribution and electron‐rich structure formation, thus enhancing light absorption, accelerating charge separation, and improving H_2_O_2_ formation.

In another case, nitrogen vacancies and oxygenophilic Co nanoclusters were introduced into carbon nitride by Zheng's group, leading to the formation of CoO_x_‐NvCN, giving CoO_x_‐NvCN with a distinctive presence of unpaired electrons, thereby establishing an electron‐rich structure.^[^ ^134^ ^]^ Such an intriguing electronic architecture is elegantly corroborated by EPR (Figure 5f), providing a compelling visual representation of enhanced electronic properties. As exhibited in Figure 5g,h, the H_2_O_2_ generation of CoO_x_‐NvCN is 244.8 µm h^−1^ in pure water under simulated visible light irradiation, which is ≈12.1 times that of BCN and 2.5 times that of NvCN, which might be attributed to the manifestation of enhanced active sites. To take a deep insight into the relationship between active sites and H_2_O_2_ generation, the charge density difference diagrams between the •OOH and BCN, NvCN, and CoO_x_‐NvCN are explored, and a lowest adsorption energy and a more visually apparent redistribution of charge with the •OOH group were observed for CoO_x_‐NvCN, implying the strengthened adsorption of •OOH group on CoO_x_‐NvCN for H_2_O_2_ generation. Accordingly, it is claimed that the increased active sites with defect engineering might promote the surface adsorption capacity, which is advantageous for the enhanced H_2_O_2_ photosynthesis in the practical application. Intriguingly, the electronic structure of the substrate was optimized through introducing N defects, which is advantageous for the enhanced charge separation efficiency and improved photocatalytic performance.

**Figure 6:**
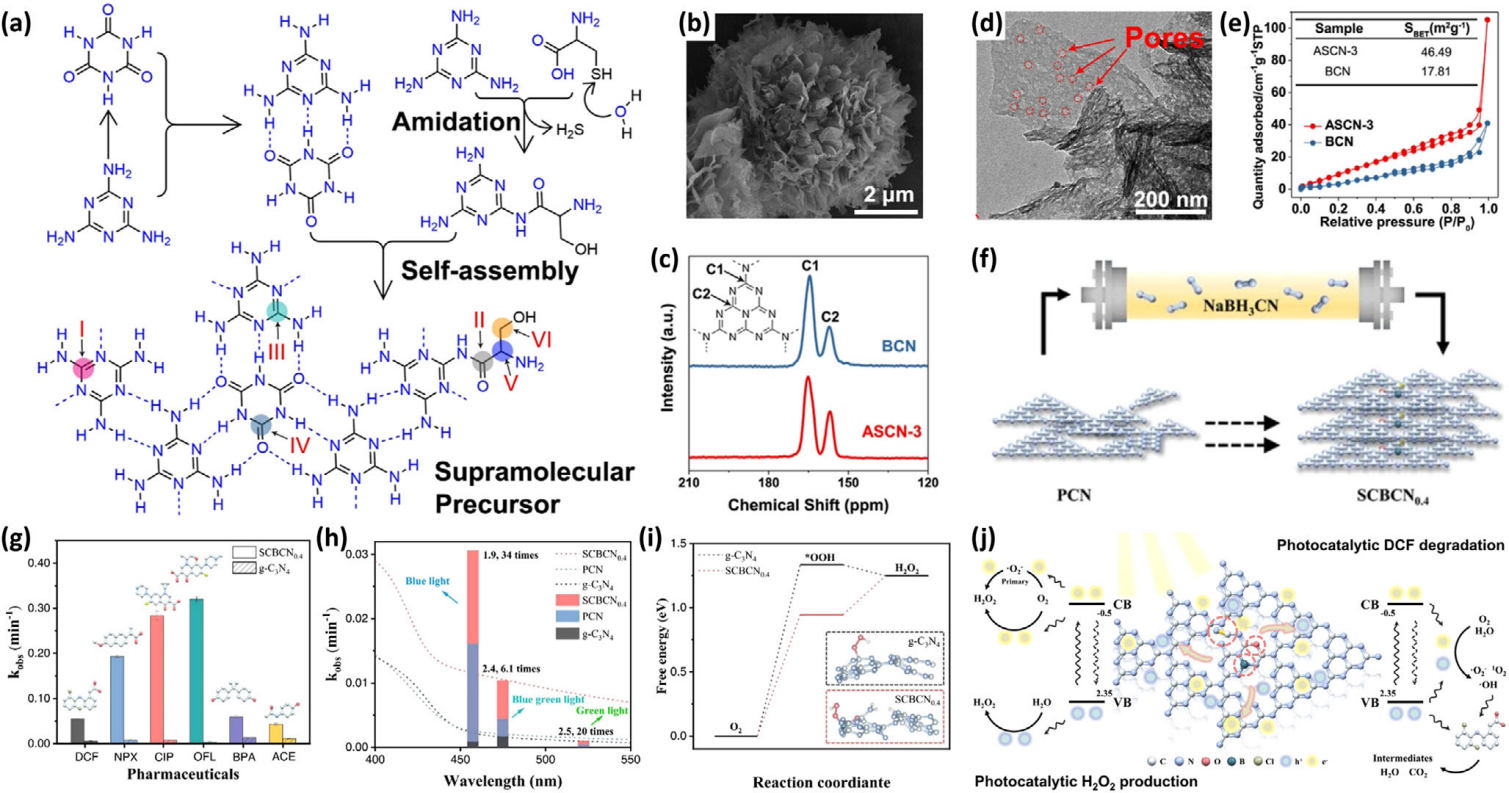
a) Fluid shear stress schematic diagram, b) SEM image of ASCN‐3, c) the solid‐state ^13^C NMR spectra of ASCN‐3 and BCN, d) TEM image of ASCN‐3, e) N_2_ adsorption–desorption isotherms of ASCN‐3 and BNA. Reproduced with permission.^[^ ^117^ ^]^ Copyright 2023, American Chemical Society. f) Schematic of SCBCN_0.4_ syntheses from PCN, g) O 1s XPS spectra of PCN, SCBCN_0.4_, and SBCN_0.4_, h) solid‐state ^11^B NMR spectrum of SCBCN_0.4_, i) calculated Gibbs free energy diagrams for ORR over g‐C_3_N_4_ and SCBCN_0.4_, j) mechanism over SCBCN_0.4_. Reproduced with permission.^[^ ^135^ ^]^ Copyright 2022, Elsevier.

Additionally, defect engineering over carbon nitride might result in an increased specific surface area, which also correlates with enhanced adsorption capability and H_2_O_2_ photosynthesis. An excellent example is the usage of fluid shear stress to regulate self‐assembly to obtain acanthosphere‐like supramolecular precursors (AS), and the precursors were treated in a muffle furnace to yield ASCN‐x composed of ultrathin nanosheets assembled with nitrogen vacancies and carbonyl modification (**Figure** **6**a,b).^[^ ^117^ ^]^ Specifically, the application of fluid shear stress is achieved through the combination of melamine and L‐cysteine, in which L‐cysteine can promote the hydrolysis of melamine, thus preventing the stacking of interlayer π─π bonds and resulting in the formation of spiky spherical bundles. As shown in Figure 6c, the solid‐state ^13^C NMR peaks of ASCN‐3 are similar to those of BCN, whereas the higher signal intensity ratio of C1 to C2 in ASCN‐3 indicates the existence of nitrogen vacancies located near C_3_N. As revealed in Figure 6d,e, TEM images and N_2_ adsorption–desorption isotherm measurements were conducted. Intriguingly, ASCN‐3 indeed has more cavities distributed throughout the lamellar nanostructure, along with a promoted specific surface area of 46.49 m^2^ g^−1^, which is approximately three times larger than that of BCN (17.81 m^2^ g^−1^). Necessarily, the larger specific surface area and abundant active sites of ASCN‐3 might result in a stronger adsorption capacity, which in turn leads to ideal performance in photocatalytic H_2_O_2_ production. This work pioneers a convenient fluid shear strategy to prepare thin nanosheets by exfoliating the stacked structure coupled with photocatalysis, thereby increasing the specific surface area, enhancing the adsorption capacity, and fully utilizing photogenerated electrons, holes, and atoms for redox reactions. Notably, the carefully inserted N vacancies and carbonyl groups lead to electron redistribution and inhomogeneity, which promotes the spatial separation of charge carriers and generates dual active sites for surface adsorption and redox reaction.

In another study, the relationship between defect engineering, electronic structure, and adsorption energy is explored from another perspective by Yu's group.^[^ ^135^ ^]^ Sodium borohydride‐modified carbon nitride (SCBCN_x_) was synthesized through thermal treatment, which not only introduced cyano defects but also modified PCN with boron and oxygen elements (Figure 6f). The modification of B and O elements was confirmed by high‐resolution O 1s XPS spectra and solid‐state ^11^B NMR spectra (Figure 6g,h). In the O 1s XPS spectrum of PCN, there is only one peak from H_2_O adsorbed on the catalyst surface, while SCNCN_0.4_ has two new peaks, like C─O or N─C─O at 531.2 eV, and C─OH at 533.5 eV. Furthermore, the appearance of two peaks at 16.8 and −3.2 ppm in the solid‐state ^11^B NMR spectrum demonstrates the successful introduction of the B element into the structure. The increased specific surface area of SCBCN_0.4_ leads to a higher H_2_O_2_ production, which is a manifestation of its excellent performance. As exhibited in the DFT‐calculated 2e^−^‐ORR Gibbs free energy diagram, the energy barrier for •OOH on SCBCN_0.4_ is significantly reduced compared to g‐C_3_N_4_ owing to the implementation of defect engineering, implying the easier reaction pathway for H_2_O_2_ photosynthesis (Figure 6i). Comprehensively, cyano defects, B elements, and O elements inside SCBCN_0.4_ with a large specific surface area can act as hubs for the separation of electrons and holes, thus accelerating the directional migration of electrons. This is not only a manifestation of the generation of abundant active sites but also the source of stronger adsorption capacity and a logical explanation for the higher yield of H_2_O_2_ (Figure 6j). Crucially, an electronic structure conducive to photocatalytic H_2_O_2_ production was constructed through the strong electron‐withdrawing effect of ─C ≡ N group and doping modulation. This research showed the possibility of improving the electronic structure and adsorption energy through defect engineering, and provided an innovative method for fabricating triple‐strategy modified g‐C_3_N_4_ for efficient H_2_O_2_ production.

In an eye‐catching study, Zhao et al.^[^ ^90^ ^]^ introduced electron‐withdrawing cyanoamino groups and coordination interactions between Na and pyridinic nitrogen into the carbon nitride framework by molten‐salt treatment with sodium cyanate, thereby altering the energy distribution of the carbon nitride skeleton (PCN‐NaCA‐2). The structural evolution enhanced the photon absorption capability and delayed the emission of charge carriers. It also led to strong interactions between surface charges and molecular oxygen. To further explore the performance of PCN‐NaCA‐2 in large‐scale reactions, a continuous tandem micro‐batch reactor was employed. The H_2_O_2_ generation performance of PCN‐NaCA‐2 was 24.6 times that of PCN, and the feasibility of practical application over PCN‐NaCA‐2 was confirmed. This study employed defect engineering to adjust and optimize the electronic structure of carbon nitride and utilized large‐scale reaction simulations to mimic practical applications, paving the way for the future industrial development of defective carbon nitride. In another case, Wu's group utilized the synergistic effect of argon gas cracking and supramolecular self‐assembly to synthesize carbon nitride containing nitrogen vacancies in a one‐step process.^[^ ^73^ ^]^ The introduction of nitrogen vacancies significantly adjusted the electronic structure, promoted the separation of photogenerated electron‐hole pairs, and enhanced the adsorption of H^+^ and O_2_. In further consideration of the industrial application over defective carbon nitride, a two‐step tandem reaction system based on photocatalytic H_2_O_2_ production and propylene epoxidation was constructed and scaled up, paving a promising way for the industrial development of defective carbon nitride in large‐scale applications.

In summary, from the perspective of adsorption, defect engineering can modulate electronic structure and increase the specific surface area of materials, thus leading to ideal physical‐chemical properties and more reactive sites formation. Consequently, the energy barrier for the production of H_2_O_2_ becomes more easily achievable. This strategy not only provides an effective approach to enhance photocatalytic performance by improving it from 2D‐thermodynamics (reducing reaction energy barriers) and kinetics (accelerating the adsorption‐reaction process), but also paves a new research direction for designing efficient and stable photocatalytic materials.

### 3.3 Pathway Regulation for H_2_O_2_ Photosynthesis

Interestingly, defect engineering has notably played a remarkably satisfactory role in pathway regulation and selectivity promotion for effective H_2_O_2_ production, which is advantageous for the sustainable solar‐to‐chemical conversion. Therefore, characterizations like electrochemical measurements and DFT calculations have been applied to explore the instrinic reaction pathway in the photocatalytic reaction.

**Figure 7:**
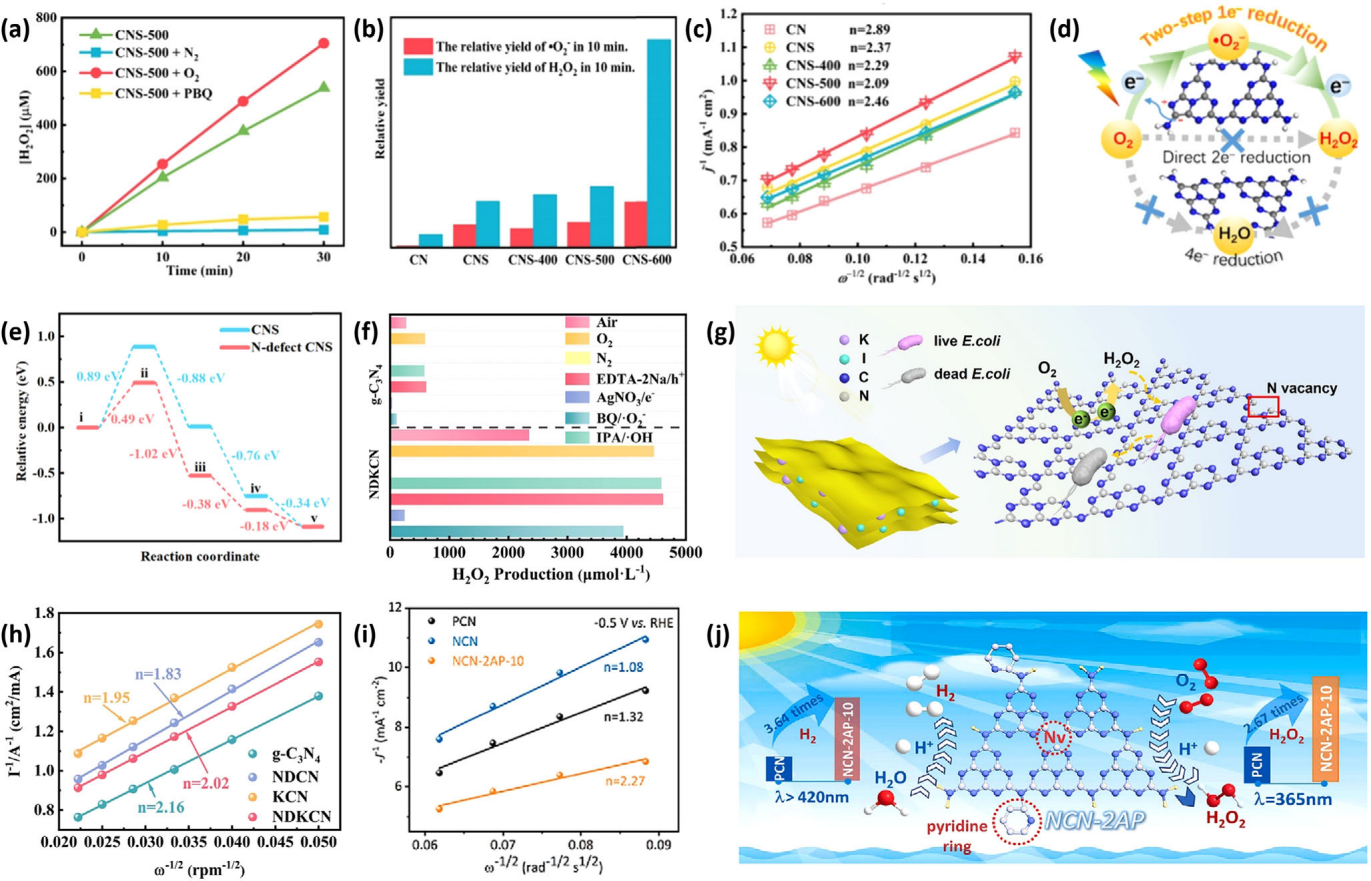
a) Free radical trapping experiments over CNS‐500, b) relative yields of H_2_O_2_ and •O_2_^−^ of CNS‐500, c) Koutecky–Levich plots of the ORR data measured by RRDE analysis for CN, CNS, CNS‐400, CNS‐500, and CNS‐600, d) mechanism of H_2_O_2_ production by CNS‐x, e) the relative energies of each step of H_2_O_2_ production for CNS and N‐defect CNS. Reproduced with permission.^[^ ^136^ ^]^ Copyright 2021, Elsevier. f) Free radical trapping experiments over g‐C_3_N_4_ and NDKCN, g) mechanism of H_2_O_2_ production by NDKCN, h) RRDE analysis. Reproduced with permission.^[^ ^137^ ^]^ Copyright 2024, Elsevier. i) RRDE analysis, j) mechanism of H_2_O_2_ production by PCN and NCN‐2AP‐10. Reproduced with permission.^[^ ^2^ ^]^ Copyright 2022, Elsevier.

For instance, in an elegant study, a series of nitrogen‐deficient carbon nitride‐based photocatalytic materials (CNS‐x) was synthesized by Liu et al.^[^ ^136^ ^]^ through a multi‐step calcination procedure, among which CNS‐500 exhibited an outstanding performance 18 times higher than that of pristine CN in the presence of a sacrificial agent (**Figure** **7**a). In order to explore the intrinsic mechanism of CNS‐500, radical scavenging experiments were conducted (Figure 7b), and it is indicated that O_2_ and •O_2_^−^ are essential components in the photocatalytic process. Further investigation was carried out through the Koutecky–Levich analysis of ORR data obtained by rotating ring‐disk electrode (RRDE) measurements (Figure 7c), showing that the average electron transfer number (n) of CNS‐500 (2.09) is closer to 2 as compared to that of CN (2.89), which is a sign of a more favorable two‐electron pathway for H_2_O_2_ production over CNS‐500. Given the conclusions drawn from the radical scavenging experiments and the results of the electron transfer number, these two lines of evidence point to a two‐step single‐electron ORR pathway for H_2_O_2_ production from O_2_, which involves the following steps: O_2_ + e^−^ → •O_2_^−^ and •O_2_^−^ + 2H^+^ + e^−^ → H_2_O_2_ (Figure 7d). Interestingly, a more specific and precise mechanism for H_2_O_2_ production was proposed through DFT calculations (Figure 7e). The excitation of visible light endows CNS‐500 with energy, enabling the separation of e^−^ and h^+^ i). Subsequently, the sacrificial agent is oxidized by h^+^ to produce protons ii), and O_2_ is adsorbed onto the surface of the as‐synthesized CNS‐500 iii). Following this, the adsorbed O_2_ reacts with e^−^ to form •O_2_^−^, which subsequently reacts with protons to form •OOH iv). Finally, •OOH reacts with another proton to produce H_2_O_2_ v). This mechanism is also known as the proton‐coupled electron transfer (PCET) pathway, which is widely recognized as one of the most fundamental and extensively studied mechanisms in photocatalysis. This work provides a comprehensive and in‐depth exploration of the mechanism underlying H_2_O_2_ production by defective carbon nitride photocatalysts, employing a multifaceted and multidimensional approach to elucidate the intricacies of the process.

Coincidentally, in another study, radical scavenger experiments were carried out in a more comprehensive manner.^[^ ^137^ ^]^ The characteristic feature of NDKCN is the simultaneous introduction of nitrogen vacancies along with iodine and potassium ions. The performance of NDKCN under different atmospheres (air, O_2_, and N_2_) shows that O_2_ is indispensable for the H_2_O_2_ photosynthesis (Figure 7f), confirming that NDKCN and g‐C_3_N_4_ are indeed involved in the ORR process rather than the WOR process. Further investigations have been carried out to explore the instrinic reaction pathway, like direct ORR (one‐step two‐electron pathway) or indirect ORR (two‐step single‐electron pathway). Subsequently, both NDKCN and g‐C_3_N_4_ were affected after capturing e^−^. However, upon capturing •O_2_^−^, it was found that the activity of g‐C_3_N_4_ was significantly reduced, while NDKCN remained largely unaffected, implying an indirect ORR process over g‐C_3_N_4_ and a direct ORR process over NDKCN (Figure 7g). Furthermore, it is shown that the value for NDKCN (n = 2.02) is closer to 2, which further corroborates the reliability of the aforementioned conclusions (Figure 7h). Above all, this study convincingly demonstrated that a direct ORR mechanism could also achieve remarkably satisfactory photocatalytic performance, highlighting its potential as an efficient pathway for H_2_O_2_ production. In the following example, nitrogen vacancies were introduced while simultaneously incorporating pyridine rings to expand the π‐conjugated system (NCN‐2AP‐10),^[^ ^2^ ^]^ thus effectively enhancing the photocatalytic activity by broadening the light absorption range and improving charge separation efficiency. The average electron number of NCN‐2AP‐10 (n = 2.27) is closer to 2 as compared to PCN and NCN (1.08 and 1.32, respectively), indicating that NCN‐2AP‐10 has significantly enhanced ORR selectivity (Figure 7i). Considering the exceptionally strong •O_2_^−^ signal for NCN‐2AP‐10, the two‐step single‐electron pathway is considered a highly plausible and well‐supported inference (Figure 7j). Specifically, the introduction of pyridine rings and nitrogen vacancies remarkably enhances the separation efficiency of photogenerated electrons and holes, which in turn effectively promotes the selectivity for the two‐step single‐electron pathway and accelerates the production of H_2_O_2_, paving a novel pathway for the design of efficient solar conversion materials.

**Figure 8:**
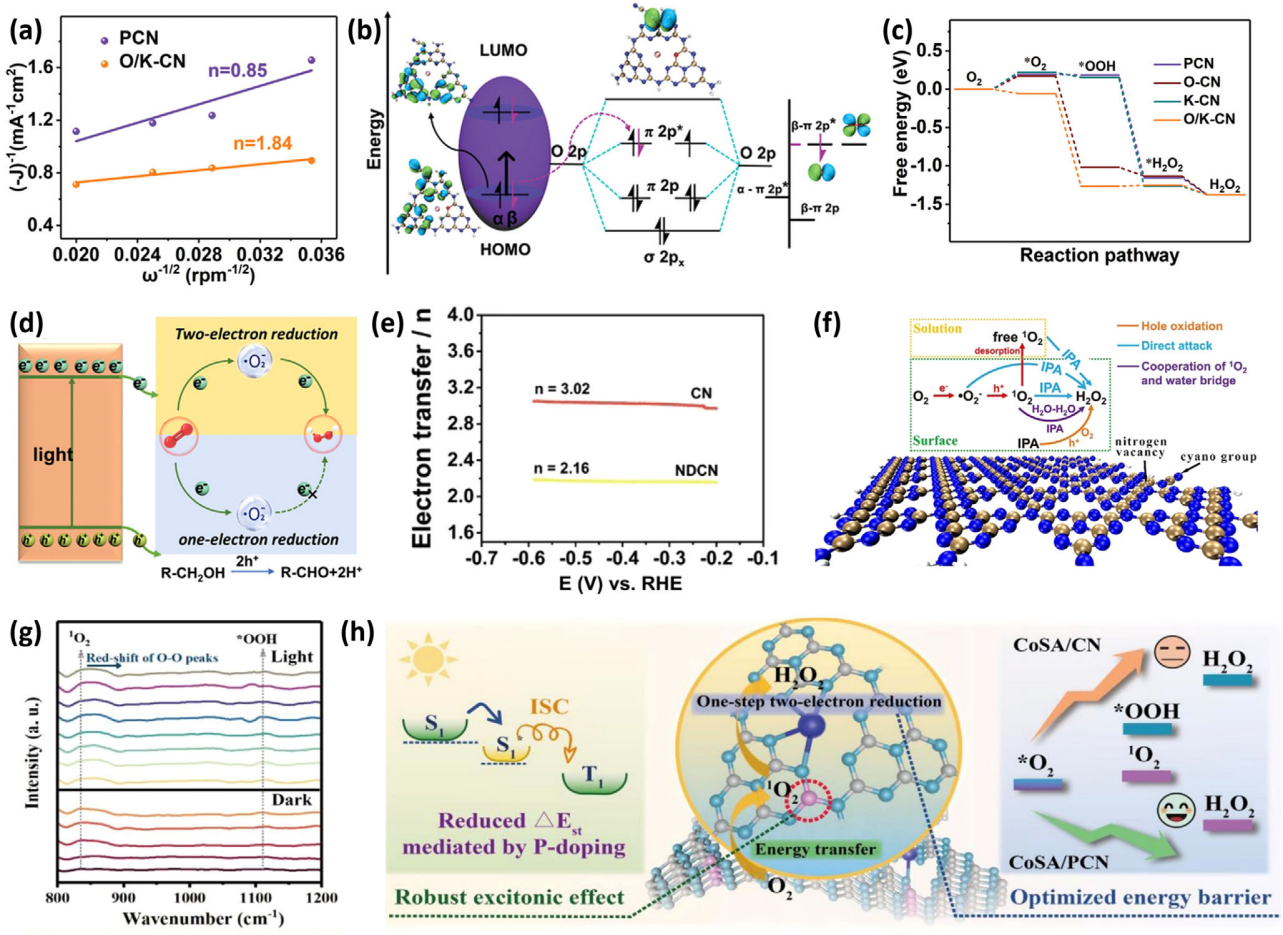
a) RRDE analysis, b) mechanism of electron migration over O/K‐CN after photoexcitation, c) free energy diagrams of samples, d) reaction mechanism for H_2_O_2_ production by O/K‐CN. Reproduced with permission.^[^ ^41^ ^]^ Copyright 2022, Wiley‐VCH. e) RRDE analysis, f) reaction mechanism for H_2_O_2_ production over NDCN. Reproduced with permission.^[^ ^5^ ^]^ Copyright 2021, American Chemical Society. g) In situ diffuse reflectance infrared Fourier transform spectroscopy (DRIFTS) spectra of CoSA/PCN, h) reaction mechanism for H_2_O_2_ production by CoSA/CN and CoSA/PCN. Reproduced with permission.^[^ ^138^ ^]^ Copyright 2023, Elsevier.

Logically, in the ORR process for photocatalytic H_2_O_2_ production, the primary step is the activation of O_2_. For instance, the introduction of dual heteroatoms into carbon nitride (O/K‐CN) has been developed by Ao's group,^[^ ^41^ ^]^ and the strengthened response signal of •O_2_^−^ from the EPR and the closer value n for O/K‐CN (1.84) to 2 for demonstrated the existence of a two‐step single‐electron ORR (**Figure** **8**a). In this case, the oxygen adsorption capacity was revealed through DFT calculations (Figure 8b), with calculated α‐HOMO, α‐LUMO, β‐HOMO, and β‐LUMO models of O/K‐CN. When O/K‐CN is excited by light, the electron transition in the α‐spin orbital forms the α‐LUMO model configuration. Conversely, the electron transition in the β‐spin orbital to the π^*^ orbital of the O_2_ molecule successfully activates O_2_. The advantage of defect engineering is clearly demonstrated in the free energy diagram, where only O/K‐CN exhibits a lower energy barrier among the four samples (Figure 8c). As shown in Figure 8d, the possible mechanism for H_2_O_2_ production by O/K‐CN and PCN is illustrated, which also well explains the reason why O/K‐CN has a higher H_2_O_2_ yield as compared to PCN. This thought‐provoking study provides in‐depth insights into the formation mechanism of H_2_O_2_ in the two‐electron ORR for H_2_O_2_ production by defect‐type photocatalysts.

Uniquely, Luo et al.^[^ ^5^ ^]^ conducted an in‐depth mechanistic study on the photocatalytic H_2_O_2_ production over nitrogen‐deficient carbon nitride (NDCN), making a significant contribution to the understanding of the H_2_O_2_ generation pathway. The ORR reaction mechanism for NDCN has been proposed, with the electron transfer number n (2.16) calculated from RRDE data being close to 2 (Figure 8e). Given that the valence band (VB) position of NDCN (+1.69 V) is not more positive than the OH^‐^/•OH potential (+2.4 V), it is proposed that •OH is more likely to be generated through the decomposition of H_2_O_2_ (H_2_O_2_ + e^−^ → •OH + OH^−^). Therefore, the radicals involved in the production of H_2_O_2_ are •O_2_^−^ and ^1^O_2_. As we know, O_2_ is converted into H_2_O_2_ via •O_2_^−^. The absence of ^1^O_2_ EPR signals after the addition of a •O_2_^−^ quencher leads to the inference that ^1^O_2_ is generated from •O_2_^−^ (O_2_ → •O_2_^−^ → ^1^O_2_ → H_2_O_2_). Upon the introduction of ^1^O_2_ quencher, the consumption of IPA is markedly diminished, thereby suggesting that ^1^O_2_ is indeed one of the reactive species responsible for the oxidation of IPA. Integrating time‐dependent density functional theory (TDDFT) simulations, DFT calculations, and rational analyses, five possible pathways for H_2_O_2_ production have been identified as follows. A) Due to photoexcitation, the β‐electron on the nitrogen vacancy at the surface of NDCN is excited. The methylene hydrogen of IPA is provided to the nitrogen atom near the nitrogen vacancy. O_2_ forms •O_2_^−^ with the excited electron. Subsequently, the electron from IPA is transferred to NDCN, converting •O_2_^−^ to O_2_^2−^. Meanwhile, IPA provides a proton (H), forming HO_2_^−^. Finally, HO_2_^−^ combines with the H provided to the nitrogen atom to form H_2_O_2_. B) The surface •O_2_^−^ directly reacts with two H from IPA to form H_2_O_2_. C) The surface singlet oxygen (^1^O_2_) directly captures two H to form H_2_O_2_. D: After capturing one H from IPA, ^1^O_2_ indirectly obtains the second H through a bridge formed by two H_2_O molecules to form H_2_O_2_. E: Free ^1^O_2_ in the solvent forms H_2_O_2_. Based on the above research, among these five pathways, one is driven by h^+^, one is driven by •O_2_^−^, and three are driven by ^1^O_2_, demonstrating that these three types of radicals all play important roles in the reaction process (Figure 8f). This groundbreaking and meticulously executed study provides an exhaustive and highly detailed examination of the diverse pathways involved in the production of H_2_O_2_. It meticulously elucidates the multifaceted roles of the various reactive radicals present in the reaction mechanism, while also highlighting the indispensable and crucial role of ^1^O_2_ in driving and facilitating the overall reaction process. In another study, a comprehensive approach combining calculations and characterizations was employed to demonstrate the existence and role of ^1^O_2_.^[^ ^138^ ^]^ As shown in Figure 8g, the in situ DRIFTS spectra of CoSA/PCN (CoSA anchored and P‐doped CN) for photocatalytic H_2_O_2_ production vividly illustrate that O_2_ is activated by energy transfer to form ^1^O_2_ upon light irradiation. Nevertheless, in the next stage after the activation of O_2_ to •O_2_^−^, it does not conventionally form •OOH, but rather activates to ^1^O_2_ and then forms H_2_O_2_ (Figure 8h). This idea undoubtedly represents a significant shift from traditional thinking, and its rationality is substantiated by energy barriers and computational results. It advances the mechanistic understanding of H_2_O_2_ production via photoexcitation in carbon nitride‐based catalysts with defect engineering.

Based on the above demonstrations and analyses, defect engineering has endowed carbon nitride with the initiative to choose, which refers to the selection of the ORR pathway. Given that the production of H_2_O_2_ primarily occurs via the ORR pathway, the conversion of O_2_ to H_2_O_2_ is facilitated by the initiative brought by defect engineering, which is undoubtedly highly satisfactory to all. It can be seen that the promotion of H_2_O_2_ production by defect‐engineered C_3_N_4_ mainly originates from the alteration of charge density, which can be evidenced by electrochemical measurements and DFT calculations. Intriguingly, the adsorption and activation of reactants and the formation of intermediates can be greatly enhanced, thereby selectively promoting the generation of oxygen active species and realizing efficient H_2_O_2_ production for practical applications.

## 4 In Situ Characterization Techniques

Defective carbon nitride plays a vital role in solar‐to‐chemical conversion, in which the in‐depth exploration into the synthetic procedure, the proper chemical structure, and the enhanced performance, as well as the further applications via tuning structures and modulating properties. Considering the real‐time observation and the manipulation of structures, the utilization of in situ characterizations is significant and predominant, especially referring to the existing reactive intermediates during the H_2_O_2_ photosynthesis as time going. In this section, the in situ characterization methods can be divided into two parts, including the synthetic characterization into the materials and the structure variation (*e.g*., in situ UV–vis spectroscopy, in situ X‐ray absorption spectroscopy (XAS), in situ X‐ray diffraction (XRD), in situ X‐ray photoelectron spectroscopy (XPS)) and the detailed reaction process with the detection of intermediates (e.g., in situ diffuse reflectance infrared Fourier transform (DRIFT) spectroscopy, in situ Raman spectroscopy, in situ electron paramagnetic resonance (EPR) spectroscopy).^[^ ^139^, ^140^, ^141^, ^142^, ^143^, ^144^, ^145^, ^146^, ^147^ ^]^

### 4.1 In Situ Methods for Structural Evolution

Considering the design and synthesis of defective carbon nitride, diverse in situ characterizations have been adopted to explore molecule structure, the surface chemical bonding, internal electronic structure, the band structure variation with the changes in external conditions, *etc*.^[^ ^87^, ^148^, ^149^ ^]^ For instance, XRD has been widely utilized to detect the phases, crystallinity, and preferred orientation of the synthesized samples, while in situ XRD is introduced to investigate the structural change of the molecular structure with the environmental conditions changing (like the temperature, the atmosphere, *etc*.^[^ ^150^, ^151^ ^]^). Taking another example, XAS is considered as a novel element‐specific analysis characterization under a wide range of experimental conditions, and the corresponding is in situ XAS approach enables new experimental designs to directly track changes in structure and composition, which seems more careful and persuasive.^[^ ^152^, ^153^ ^]^ Therefore, in this section, in situ characterizations utilized in the structure variation investigation during the fabrication or the reaction has been concluded and discussed clearly.

**Figure 9:**
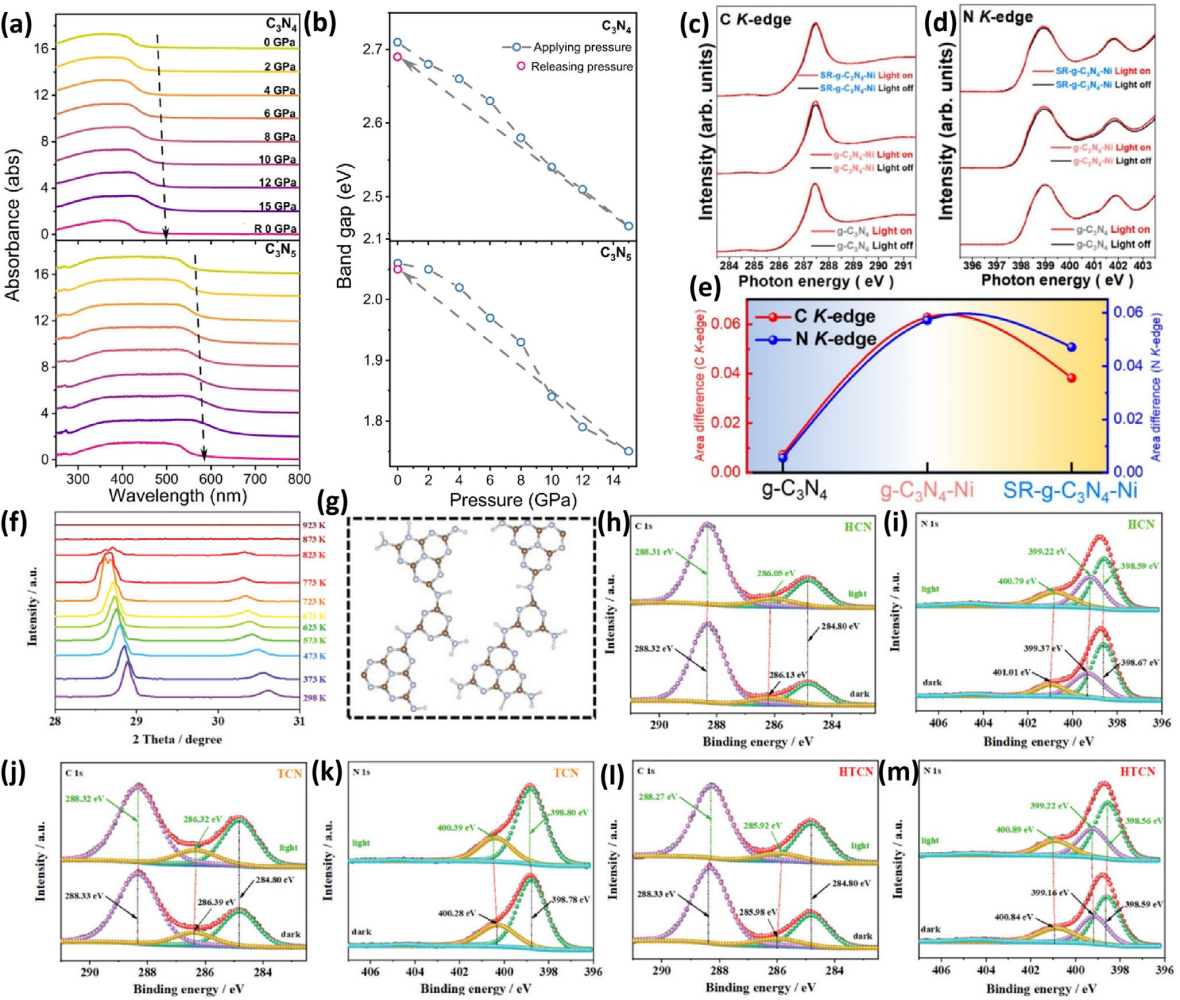
a,b) In situ pressure‐dependent UV–vis absorption spectra and bandgap variation with pressure of C_3_N_4_ and C_3_N_5_. Reproduced with permission.^[^ ^154^ ^]^ Copyright 2023, Nature Publishing Group. c,d) In situ C K‐edge and in situ N K‐edge spectra of g‐C_3_N_4_, g‐C_3_N_4_‐Ni, and SR‐g‐C_3_N_4_‐Ni with/without visible light, e) differences in spectra for in situ XAS at C and N K‐edge. Reproduced with permission.^[^ ^155^ ^]^ Copyright 2023, American Chemical Society. f) In situ XRD spectra of CM and KCl/LiCl composites under different temperatures from 303 to 923 K, g) the calculated structure model of HTCN, In situ XPS measurement under visible light irradiation (𝜆 = 420 nm): h,i) C 1s and N 1s of HCN, j,k) C 1s and N 1s of TCN, l,m) C 1s and N 1s of HTCN. Reproduced with permission.^[^ ^156^ ^]^ Copyright 2023, Wiley‐VCH.

Under normal circumstances, UV–vis absorption spectra have normally been utilized to explore the absorption edge and band structure of photocatalysts. Following this, novel in situ UV–vis absorption spectroscopy is adopted to investigate the relationship between the change in the absorption edge, the band structure, and the environmental conditions, like the in situ pressure‐dependent UV–vis absorption can explore the relationship between the band structure and the pressure under controlled variable pressure conditions. For example, Li et al.^[^ ^154^ ^]^ prepared a defect‐engineered composite material–nitrogen‐rich triazole‐based carbon nitride (C_3_N_5_)–via a one‐step thermal polymerization method, and the synthesized C_3_N_5_ could achieve an outstanding performance with a H_2_O_2_ photosynthesis rate of 1904.75 µm h^−1^ and a 2e^−^ transfer selectivity of 92% upon the synergistic power of sunlight and ultrasonic forces, attributed to the spontaneous polarization of photogenerated charge kinetics to produce hydrogen peroxide. In this case, it can be observed that the absorption edges of C_3_N_4_ and C_3_N_5_ are red‐shifted with increasing ambient pressure, and compared to C_3_N_4_, the red‐shift behavior of C_3_N_5_ is more dramatic (**Figure** **9**a). Nevertheless, when the increased pressure is reduced back to 0 GPa, a red shift in the absorption edge is still observed compared to the absorption edge before the pressure change. That is to say, the bandgaps of both materials become smaller under external force, thereby enhancing charge migration (Figure 9b, the band gap of C_3_N_4_ decreases from 2.71 to 2.69 eV, while the band gap of C_3_N_5_ drops from 2.06 to 2.05 eV). This result rationally explains the reasons for the higher H_2_O_2_ generation and faster electron migration of C_3_N_5_. Interestingly, this study innovatively employed in situ pressure‐dependent UV–vis absorption spectra under variable pressure to elucidate the relationship between pressure and the band structure, thereby explaining the reason for the increased charge migration rate under external force.

Considering the electronic structure of the synthesized catalysts, X‐ray absorption spectroscopy (XAS) is regarded as one of the most practical methods for investigating the internal electronic structure, while in situ XAS spectra are employed to explore the excitation of internal charges and the modulated electronic structure in the material. For example, Chou's group^[^ ^155^ ^]^ utilized single‐atom nickel (Ni) terminators to coordinate with the heptazine units of g‐C_3_N_4_, forming a carbon nitride with carbon vacancies and introduced Ni atoms (g‐C_3_N_4_‐Ni), in which in situ XAS was adopted to explore the electronic change and charge separation process of the structure. As shown in Figure 9c,d, the peak intensity of the C K‐edge near 287.5 eV increases for g‐C_3_N_4_‐Ni after illumination, indicating the generation of photogenerated hole states at the N─C─N sites in sp^2^‐hybridized carbon; the peak intensity of the N K‐edge shows a significant enhancement, which suggests an increase in the number of photogenerated N 2p π^*^ states at the pyridinic N sites located in the edge C═N─C bonds. This means that compared to g‐C_3_N_4_ and g‐C_3_N_4_‐Ni after the photocatalytic reaction underwent a structural self‐reorganization (SR‐g‐C_3_N_4_‐Ni), g‐C_3_N_4_‐Ni could generate more electrons and holes under the influence of light, in which might be a vital factor for a more efficient photocatalytic H_2_O_2_ generation. As concluded in Figure 9e, the area change of the C K‐edge in SR‐g‐C_3_N_4_‐Ni is smaller than that of the N K‐edge, implying that the covalence between C and N has changed under the photocatalytic self‐reorganization of the local atomic structure. It is noteworthy that in situ XAS can be adopted to clearly analyze the change of the internal electronic structure of the photocatalyst and its response efficiency to light, which is advantageous for understanding the capacity of a photocatalyst.

As is well known, g‐C_3_N_4_ is a 2D layered material composed of heptazine ring structural units bridged by nitrogen atoms. In another interesting study, the carbon nitride photocatalyst was designed into a form combining heptazine rings with triazine rings (HTCN) by controlling the annealing temperature, achieving stronger carrier density and photogenerated electron diffusion capability.^[^ ^156^ ^]^ The mysterious veil of HTCN's structure and internal electron transfer was unveiled by in situ XRD and in situ XPS. As exhibited in Figure 9f,g, with the annealing temperature increasing from 298 to 673 K, the peak of the (002) undergoes a negative shift, which is attributed to the effect of alkali metals. As the annealing temperature reaches 723 K, another peak of the (002) emerges while the original peak continues to shift negatively. The in situ XPS spectra of carbon nitride annealed solely with the addition of KCl (HCN), carbon nitride annealed solely with the addition of LiCl (TCN), and HTCN are presented in Figure 9h–m. Intriguingly, it is observed that under visible light irradiation, the binding energy (BE) undergoes a negative shift for all three samples, indicating enhanced electron transfer from the N atom to the C atom. On the other hand, the BE of HCN undergoes a negative shift, while that of TCN and HTCN undergoes a positive shift, indicating a reduction in the electron density around the nitrogen atoms in HTCN. This work ingeniously employs in situ XRD and in situ XPS to investigate the internal electronic structure, like the electron density of elements and the electron transfer inside defective carbon nitride, which provides guidance in the design and detection into the synthesis of such materials.

Above all, the importance of in situ characterizations in the exploration phase of photocatalytic materials has been listed and compared, which might be an essential tool in the further development of modified carbon nitride. Moreover, the coupling of different in situ spectroscopy techniques can help to understand the electronic structure of photocatalysts more clearly. In situ XRD can deepen the understanding of the dynamic structural changes of photocatalysts during the reaction process, which is not achievable with conventional XRD. In situ UV–vis absorption spectroscopy can investigate the relationship between light absorption properties and environmental conditions, which conventional UV–vis absorption spectroscopy cannot clarify under dynamic conditions. In situ XPS and in situ XAS can track the chemical state changes and monitor the local structural evolution during material synthesis or during the reaction, providing detailed insights into catalyst formation and interfacial changes, which are challenging to be achieved with conventional XPS and XAS under equilibrium conditions. In the context of the prevalence of defect engineering, in situ characterization techniques have made significant contributions to the study of molecular structure and internal electronic structure, as well as the band structure variation with the changes in external conditions. In the future, in situ characterizations integrated with artificial intelligence might be a more powerful means to study structural variation clearly, thereby providing a deeper insight into the physicochemical properties of materials.

### 4.2 In Situ Techniques of Reaction Pathway Identification

In addition to the development in the structure observation and investigation, in situ characterizations can also act as a compass for exploring the existing intermediates, the reactive species transformation, and the detailed reaction pathway. Therefore, various in situ characterizations have been employed to monitor the generation of radicals or intermediates and reveal the reaction mechanism during the photocatalytic process. For example, in situ DRIFTS can track the formation and changes of reaction intermediates in real time;^[^ ^157^, ^158^ ^]^ in situ EPR provides a means to detect short‐lived radicals produced in photocatalysis;^[^ ^159^, ^160^ ^]^ and in situ Raman spectroscopy helps giving detailed information about existing intermediates and surface chemical bonds of the catalyst.^[^ ^161^, ^162^ ^]^Additionally, a combination utilization of these techniques might offer a comprehensive perspective for understanding the real intermediate formation process and product formation on the catalyst surface, thus leading to a better understanding over the nature of the reaction and providing an accurate guidance for future scientific research.

**Figure 10:**
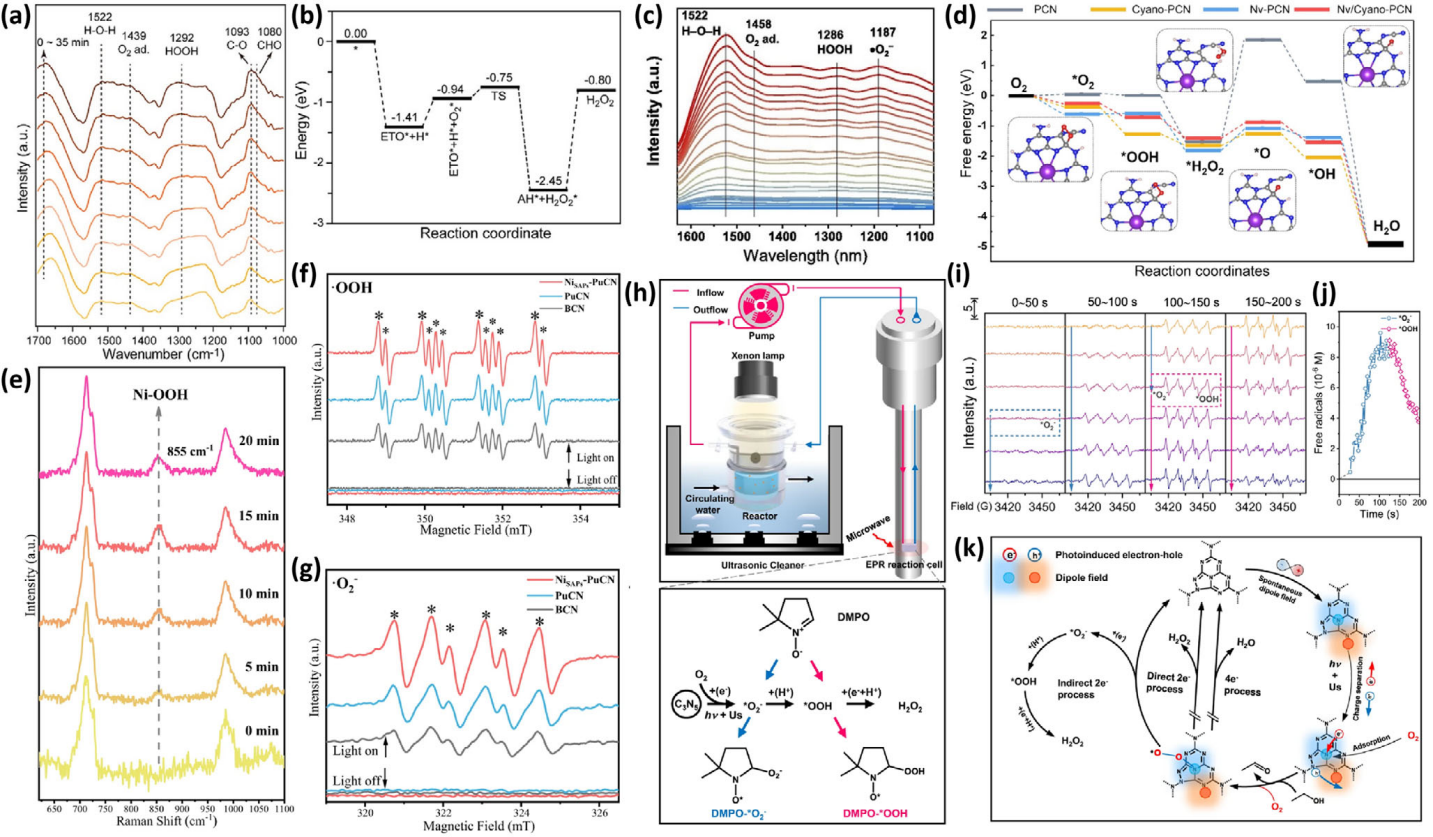
a) In situ DRIFT spectra during photocatalytic H_2_O_2_ production of BCN‐450, b) potential energy surface of the reaction between ETOH and O_2_ to form H_2_O_2_ on BCN, where AH represents acetaldehyde. Reproduced with permission.^[^ ^163^ ^]^ Copyright 2024, American Chemical Society. c) In situ DRIFT spectra during H_2_O_2_ photosynthesis over Nv/Cyano‐PCN, d) free energy diagrams of ORR steps on various models. Reproduced with permission.^[^ ^108^ ^]^ Copyright 2024, Elsevier. e) In situ Raman spectra of NiSAPs‐PuCN recorded during the photoreaction in O_2_ saturated aqueous solution, f,g) EPR signals of •OOH and •O_2_^−^ of the samples in the presence of 5,5‐dimethyl‐1‐pyrroline (DMPO). Reproduced with permission.^[^ ^164^ ^]^ Copyright 2023, Nature Publishing Group. h) In situ EPR system and principle of DMPO capturing •O_2_^−^ and •OOH, i) in situ EPR spectra and j) concentration and lifetime of different free radicals during H_2_O_2_ production, k) proposed reaction mechanism. Reproduced with permission.^[^ ^154^ ^]^ Copyright 2023, Nature Publishing Group.

Generally speaking, FT‐IR spectra are an important characterization technique for studying the functional group composition of catalysts. Therefore, in situ DRIFT spectra has been adopted to reveal the existing radicals during the photosynthetic process, thereby identifying the pathways for H_2_O_2_ generation. Taking an example, a defective carbon nitride doped with boron atoms (BCN‐450) was designed by Jiang's group,^[^ ^163^ ^]^ in which the frustrated Lewis pairs (FLPs) formed in this material effectively activate ETOH through a “push‐pull” mechanism, thereby enhancing the production of H_2_O_2_. As shown in **Figure** **10**a, in situ DRIFT spectra were employed to investigate the H_2_O_2_ formation from O_2_ and ETOH on BCN under visible light irradiation. The characteristic peaks of the HOOH (1292 cm^−1^) and the CHO (1080 cm^−1^) increased in intensity with the reaction time. The former is attributed to the symmetric bending of the two O─H bonds in HOOH, while the latter is due to the bending vibrational mode of aldehydes, indicating the formation of H_2_O_2_ and aldehyde compounds under visible light irradiation. Additionally, it can be confirmed that the adsorption of ETOH on the FLPs of BCN‐450 spontaneously dissociates into ETO^*^ and H^*^, and simultaneously, the transfer of two H atoms to O_2_ occurs on BCN, whereas it occurs sequentially on other CN‐based catalysts (Figure 10b). Subsequently, to elucidate the role of FLPs in the catalytic process, DFT calculation was employed to determine the adsorption configurations of relevant species on BCN‐450. Due to the partially frustrated nature of the FLPs on BCN‐450, the distance between the Lewis acidic B and Lewis basic N atoms changed during the catalytic reaction. In the presence of EtOH, the adsorption of O_2_ on BCN‐450 is easier than on CN, thus leading to a higher performance of BCN‐450. Based on in situ characterizations and calculations, it has been confirmed at the molecular scale that the FLPs in defective carbon nitride effectively drive the transformation of ETOH to H_2_O_2_ through a “push‐pull” mechanism. In another interesting research, as displayed in Figure 10c, Huo's group^[^ ^108^ ^]^ has also profound insights into the application of in situ DRIFT spectra, with an in‐deep insight into the reaction mechanism over nitrogen‐carbon compound with cyano groups and nitrogen vacancies (Nv/Cyano‐PCN). Under illumination, with time going, the characteristic peaks of O_2_, •O_2_^−^, and HOOH showed a significant increase, indicating that O_2_ is continuously adsorbed and transformed into the •O_2_^−^ intermediate, eventually leading to the formation of H_2_O_2_. By integrating the species identified by the characteristic peaks in the in situ DRIFT spectra and the DFT calculations, the free energy diagrams for the ORR steps on various PCN models were obtained (Figure 10d). As exhibited, Nv/Cyano‐PCN exhibited a more moderate and spontaneous two‐electron ORR process for H_2_O_2_ generation. Furthermore, a good linear relationship was found between the concentration of nitrogen vacancies and the ratio of •O_2_^−^ to H_2_O_2_, which provides fundamental knowledge for investigating the ORR mechanism of photocatalysts.

Moreover, Raman spectroscopy is highly sensitive to intermediates, and it is wise to employ in situ Raman spectra to investigate the formation and dynamics of intermediates and the pathways of photocatalytic reactions. For example, Zhang et al.^[^ ^164^ ^]^ took advantage of this sensitivity to explore the H_2_O_2_ production pathway of high‐loading Ni single‐atom photocatalysts (NiSAPs‐PuCN) under photocatalysis. As shown in Figure 10e, with time going, the O═O stretching mode in Ni─OOH becomes increasingly evident, and the presence of OOH and ·O_2_^−^ is demonstrated in Figure 10f,g. Integrating a series of characterizations, it is revealed that under light irradiation and in the presence of O_2_, the 2e^−^ ORR reaction of NiSAPs‐PuCN is further driven (Ni^*^ + O_2_ + 2e^−^ + 2H^+^ → Ni^*^ + H_2_O_2_; O_2_ → •O_2_^−^ → •OOH → H_2_O_2_). Notably, in situ Raman spectroscopy was adopted to capture the key intermediate •OOH on the Ni sites. Combined with in situ X‐ray absorption fine structure (XAFS) and theoretical calculations, the structural evolution of the Ni single‐atom active centers has been confirmed. Combined with DFT calculations, it is shown that the optimized structure and charge difference density of Ni_SAPs_‐PuCN after O_2_ adsorption can intuitively reflect the charge transfer from the Ni site to the adsorbed O_2_ end (O_1_‐Ni‐N_2_). Especially, the increase in the oxidation state of Ni, the delocalization of unpaired electrons on the Ni 3d orbitals, and the spontaneous charge transfer from Ni to the O *2p* orbitals of O_2_, all contribute to the formation of •O_2_^−^. This significantly reduced the formation energy barrier of •OOH, which is the key factor leading to the high activity and high selectivity of NiSAPs. In situ Raman spectroscopy can monitor the formation and dynamics of intermediates, while in situ XAFS can probe the local structural changes and electronic states of catalysts during the reaction process. Combining these techniques and DFT calculations can provide a comprehensive understanding of the chemical environment and reaction mechanism, showing guidance for researchers to design ideal photocatalysts and predict advancements in photocatalytic systems.

EPR can detect paramagnetic species such as free radicals and transition metal ions, but it cannot monitor the dynamic changes of these species during the reaction process. Therefore, the introduction of in situ EPR can provide real‐time monitoring of radical formation and decay, offering deeper insights into the reaction mechanisms of photocatalytic processes. For example, an EPR spectrometer was connected to an ultrasonic cleaner and a xenon lamp via a peristaltic pump to perform in situ EPR tests to monitor the intermediates during the H_2_O_2_ photosynthetic process over C_3_N_5_ (Figure 10h).^[^ ^154^ ^]^ Interestingly, upon irradiation, the characteristic peak of •O_2_^−^ appeared first, followed by the characteristic peak of •OOH with short lifetime and instability, thus being protonated to form H_2_O_2_, which is a signal of the indirect 2e^−^ ORR pathway (Figure 10i,j). Following this, a rational internal photocatalytic reaction mechanism was proposed (Figure 10k). The adsorbed O_2_ is stepwise reduced via an indirect 2e^−^ transfer pathway, with the intermediate products •O_2_^−^ and •OOH forming H_2_O_2_ on the triazole N_4_ surface, and the opposite holes are quenched by EtOH to provide sufficient electrons to balance the entire reaction. The integration of in situ EPR with advanced experimental setups enables real‐time monitoring of dynamic radical species during photocatalytic reactions, providing critical insights into the reaction mechanisms, such as the indirect 2e^−^‐ORR pathway, and offering a deeper understanding of the photocatalytic process.

The studies mentioned above highlight the significant role of in situ DRIFT spectra, in situ Raman spectroscopy, and in situ EPR in pathway identification. Intriguingly, in situ DRIFT spectra can provide clear information on organic molecules and radicals during the reaction process, making it highly suitable for exploring the reaction mechanisms. Moreover, in situ Raman spectroscopy, as a powerful technique for monitoring changes in target molecules and reflecting the vibrational and rotational modes of molecules, plays an important role in the in‐depth investigation of each reaction system. Another vital technology, in situ EPR, can provide dynamic information on radicals during the reaction process and reveal reaction mechanisms, which cannot be clarified by conventional EPR tests. In situ characterizations provide a direct route for in‐depth study of the radical pathways in photocatalytic reactions by monitoring the changes in the intensity of radical characteristic peaks over time. With the improvement of C_3_N_4_ through defect engineering, as well as the advent of new data analysis tools at our goal significantly increase the prevalence of in situ characterization in the field of photocatalysis. We deem that the importance of in situ characterizations to understand photocatalysts and photocatalytic mechanisms will further increase in the future.

For brevity, in situ characterizations have been adopted to explore the surface changes of electronic structure and surface bonding of the catalysts, observe the reaction intermediates, and investigate the active sites, thus taking a deeper insight into the reaction mechanism as well as expanding the application of the catalysts. More and more in situ strategies are introduced in recent years and play vital roles for a variety of catalysts. For instance, in situ XAS spectra, in situ XRD, and in situ XPS can explore the material structure and internal electronic structure. Furthermore, in situ DRIFT spectra, in situ Raman spectra, and in situ EPR spectra can explore the detection of intermediates and the reaction pathway. Of note, the integration of in situ methods with emerging novel techniques paves an interesting pathway for understanding H_2_O_2_ formation in an actual case.

## 5 Summary and Outlook

In this review, recent developments for photocatalytic H_2_O_2_ generation over defect‐engineering graphitic carbon nitride have been concluded. The design principles, including the synthetic strategies and function mechanism of defects, are discussed in detail, with attention in the formation process of the intrinsic structure, with a guidance from experimental data to theoretical simulations. The wide range of applications of the defect engineering strategy and the mechanism are outlined and compared, like the band structure and electronic structure modulation, surface adsorption capacity adjustment, and reaction pathway selectivity. Additionally, in situ characterizations utilized in recent studies have been discussed, with their potentials and advantages in practical applications. Of note, the existing limitations and development strengths of defective carbon nitride have been listed. It is believed that further developments over defective graphitic carbon nitride still lies in the in‐depth discovery of high‐performance H_2_O_2_ photosynthesis for industrial‐scale application with high solar energy utilization efficiency.

Defect engineering strategy can adjust band positions, modulate electronic structures, increase active sites, enhance charge separation, and significant achievements have been witnessed in recent decades. Nevertheless, several challenges remain to be addressed in the future. Ideal preparation processes and precise control conditions to accurately regulate the type, concentration, and distribution of defects are necessary. Another factor that should be considered for commercial application is the long‐term stability and recycle technology, which necessitates the exploitation of sustainable and high‐performance carbon nitride with defect engineering. That is to say, not only the simple cycling stability, the performance test within a long period like one month or large‐scale investigation, and the proficient recycling technology are both necessary. Such explorations are time‐consuming and challenging; however, necessary. In this case, machine learning can be taken into consideration to take a deep insight into the inner structure and the structural stability, thus can undoubtedly accelerate the discovery of industrializable catalysts and the understanding of the structure‐activity relationship in solar‐to‐chemical conversion.

Significant achievements in this research have been witnessed in recent decades. Nevertheless, several challenges remain to be addressed. The first factor for commercial application is the long‐term stability and the recycle technology, which necessitates the exploitation of sustainable and high‐performance carbon nitride with defect engineering. Not only the simple cycling test stability, the performance test within a long term like one month or large‐scale investigation, and the recycling technology are both necessary, characterizations after reaction should be considered. Such explorations are time‐consuming and challenging, however, necessary. In this case, machine learning can be taken into consideration to take a deep insight into the inner structure and the structural stability, thus can undoubtedly accelerate the discovery of industrializable catalysts and the understanding of the structure‐activity relationship in the solar‐to‐chemical conversion.

Additionally, ideal and efficient separation technology for H_2_O_2_ storage or the in situ utilization of H_2_O_2_ for the cascade reaction is of vital importance. However, the separation technology for H_2_O_2_ formation seems to be a bottleneck for photocatalytic systems owing to the limited solar energy conversion. Therefore, the discovery of high‐performance and high‐selectivity reactions is necessary, especially the attention should be paid to the high selectivity of the final products. Besides, the cascade reaction based on the in situ H_2_O_2_ photosynthesis is diverse and meaningful, like its application in the pollutants degradation and selective oxidation, in which H_2_O_2_ can act as a necessary oxidant, thus promoting the utilization efficiency of H_2_O_2_ and avoiding the trouble of the separation process. As such, the H_2_O_2_ photosynthesis strategy can overcome the limitations of the original industrial production method of H_2_O_2_, and offers appealing technology for satisfying solar energy utilization.

Furthermore, more advanced characterization technologies explore can be applied to explore the reaction mechanisms, like in situ technology to observe the reaction intermediates and the H_2_O_2_ formation pathway. Besides, the changing process of the functional groups and defects can be detected clearly during the whole reaction, which is beneficial for the large‐scale investigation of defects engineering carbon nitride. Overall, benefiting from the recent advances in the experimental and theoretical research over the H_2_O_2_ photosynthesis over defects‐engineering carbon nitride, we hope that more reliable technology and mechanism research can be explored in the future.

## Conflict of Interest

The authors declare no conflict of interest.
